# Salicylic acid activates poplar defense against the biotrophic rust fungus *Melampsora larici‐populina* via increased biosynthesis of catechin and proanthocyanidins

**DOI:** 10.1111/nph.15396

**Published:** 2018-08-31

**Authors:** Chhana Ullah, Chung‐Jui Tsai, Sybille B. Unsicker, Liangjiao Xue, Michael Reichelt, Jonathan Gershenzon, Almuth Hammerbacher

**Affiliations:** ^1^ Department of Biochemistry Max Planck Institute for Chemical Ecology Hans‐Knöll‐Straße 8 07745 Jena Germany; ^2^ School of Forestry and Natural Resources Department of Genetics Department of Plant Biology University of Georgia Athens GA 30602 USA; ^3^ Key Laboratory of Forest Genetics and Biotechnology Co‐Innovation Center for Sustainable Forestry in Southern China College of Forestry Nanjing Forestry University Nanjing Jiangsu 210037 China; ^4^ Department of Zoology and Entomology, Forestry and Agricultural Biotechnology Institute University of Pretoria Private Bag X20 Pretoria 0028 South Africa

**Keywords:** benzothiadiazole (BTH), chemical defense, condensed tannins, drought stress, flavan‐3‐ols, phytohormones, *Populus nigra*

## Abstract

- Poplar trees synthesize flavan‐3‐ols (catechin and proanthocyanidins) as a defense against foliar rust fungi, but the regulation of this defense response is poorly understood. Here, we investigated the role of hormones in regulating flavan‐3‐ol accumulation in poplar during rust infection.
- We profiled levels of defense hormones, signaling genes, and flavan‐3‐ol metabolites in black poplar leaves at different stages of rust infection. Hormone levels were manipulated by external sprays, genetic engineering, and drought to reveal their role in rust fungal defenses.
- Levels of salicylic acid (SA), jasmonic acid, and abscisic acid increased in rust‐infected leaves and activated downstream signaling, with SA levels correlating closely with those of flavan‐3‐ols. Pretreatment with the SA analog benzothiadiazole increased flavan‐3‐ol accumulation by activating the MYB–bHLH–WD40 complex and reduced rust proliferation. Furthermore, transgenic poplar lines overproducing SA exhibited higher amounts of flavan‐3‐ols constitutively via the same transcriptional activation mechanism. These findings suggest a strong association among SA, flavan‐3‐ol biosynthesis, and rust resistance in poplars. Abscisic acid also promoted poplar defense against rust infection, but likely through stomatal immunity independent of flavan‐3‐ols. Jasmonic acid did not confer any apparent defense responses to the fungal pathogen.
- We conclude that SA activates flavan‐3‐ol biosynthesis in poplar against rust infection.

## Introduction

Plants evolved sophisticated chemical defense mechanisms to protect themselves from biotic and abiotic stresses. They synthesize an enormous diversity of specialized metabolites to defend against pathogens and herbivores, or to increase tolerance to abiotic stresses (Moore *et al*., 2014). Poplar trees accumulate high quantities of phenolic metabolites, such as salicinoids, proanthocyanidins (PAs, also known as condensed tannins), hydroxycinnamic acids, and monolignols in their leaves, stems, and roots (Lindroth & Hwang, 1996; Tsai *et al*., 2006; Chen *et al*., 2009). PAs and their monomeric flavan‐3‐ol building blocks (catechin, epicatechin, and gallocatechin) are major end products of the flavonoid pathway (Dixon *et al*., 2005). The biosynthesis and accumulation of PAs were shown to increase in leaves of several poplar species infected by the biotrophic rust fungi *Melampsora* spp. (Miranda *et al*., 2007; Ullah *et al*., 2017), the most destructive poplar pathogen world‐wide (Pinon *et al*., 1987). Moderately resistant poplar genotypes constitutively accumulated higher amounts of flavan‐3‐ols at the site of fungal infection than susceptible genotypes did, implicating catechin and PAs as effective antifungal chemical defenses against rust infection (Ullah *et al*., 2017). These compounds also act as defenses against necrotrophic pathogens in other woody plants (Hammerbacher *et al*., 2014; Wang *et al*., 2017b). Flavan‐3‐ols are also induced in poplars by various abiotic stressors, such as ultraviolet light, high temperature, and mechanical wounding (Tsai *et al*., 2006; Mellway *et al*., 2009; Wang *et al*., 2017b). Thus, poplar responds to an array of environmental stimuli by synthesizing increased amounts of catechin and PAs in its leaves. The biosynthesis of flavan‐3‐ols in poplar is well studied (Wang *et al*., 2013; Ullah *et al*., 2017), and their regulation by the MYB–bHLH–WD40 (MBW) complex of R2R3 MYB, basic helix–loop–helix (bHLH), and WD40 transcription factors has also been reported (Mellway *et al*., 2009; Yoshida *et al*., 2015; James *et al*., 2017; Wang *et al*., 2017b). However, the role of hormones in the regulation of flavan‐3‐ol biosynthesis is largely unknown.

The phytohormones salicylic acid (SA), jasmonic acid (JA), and abscisic acid (ABA) are involved in defenses against biotic and abiotic stresses as well as in plant development (Santner *et al*., 2009; Pieterse *et al*., 2012). Typically, SA activates defense responses against biotrophic pathogens and piercing–sucking insects, whereas JA activates defenses against necrotrophs and chewing insects (Thomma *et al*., 1998). ABA enhances plant tolerance to drought stress (Cutler *et al*., 2010; Jia *et al*., 2016) and modulates plant defenses depending on the type of pathogen attack (Asselbergh *et al*., 2008; Ton *et al*., 2009). Hormonal crosstalk fine‐tunes plant defense responses against specific attackers. For example, the crosstalk between SA and JA is often antagonistic (Vlot *et al*., 2009; Pieterse *et al*., 2012).

SA can be synthesized from l‐phenylalanine via a benzoic or coumaric acid intermediate (Vlot *et al*., 2009). Phenylalanine ammonia lyase catalyzes the first reaction in this pathway, which is also the first and common enzymatic step for phenylpropanoid and flavonoid biosynthesis (Dixon *et al*., 2002; Vogt, 2010). SA is further metabolized to its *O*‐β‐glucoside (SAG) by a specific SA‐glucosyltransferase (Lee & Raskin, 1999; Song, 2006). In *Arabidopsis*, signaling downstream of SA is mostly controlled by a master regulator called NONEXPRESSOR of PR GENES1 (NPR1), which activates a large set of transcription factors and defense genes (Dong, 2004; Wang *et al*., 2006; Moore *et al*., 2011). Benzothiadiazole (BTH) is a functional analog of SA (Kunz *et al*., 1997) that can induce plant defense responses by activating the expression of defense‐related genes downstream of SA (Gorlach *et al*., 1996; Lawton *et al*., 1996; Morris *et al*., 1998; Latunde‐Dada & Lucas, 2001). JA is synthesized from the precursor molecule 12‐oxo‐phytodienoic acid (*cis*‐OPDA) in the peroxisome. Several functional groups can also be added to the JA backbone, including isoleucine, hydroxyl, carboxyl, methyl, glucosyl, and sulfate ester residues, which may be important in activation, deactivation, or transport of jasmonate signals (Fonseca *et al*., 2009; Wasternack & Hause, 2013). ABA is an isoprenoid hormone, best characterized for its role in inducing stomatal closure to reduce water loss under drought conditions (Xiong *et al*., 2006; Lobell *et al*., 2014). ABA can also trigger early defense responses against bacterial and fungal pathogens by inducing stomatal closure (Melotto *et al*., 2006; Sun *et al*., 2014) and callose deposition to block the entry and progression of invading pathogens (Ton & Mauch‐Mani, 2004; Ton *et al*., 2009).

The signaling pathways downstream of SA, JA, and ABA have been well studied in herbaceous plant species such as *Arabidopsis*, tobacco (*Nicotiana tabacum*), and rice (*Oryza sativa*) under different biotic and abiotic stress conditions. However, defense signaling of these hormones remains unclear in woody plants. For example, hyperaccumulation of SA in transgenic poplars elicits strong oxidative responses by activating many downstream targets of SA signaling without altering *NPR1* expression (Xue *et al*., 2013). This finding challenges the involvement of *NPR1* in poplar SA signaling. In poplars, defense‐related genes were upregulated by fungal infection in both compatible and incompatible interactions, but the response was faster in the latter (Miranda *et al*., 2007; Rinaldi *et al*., 2007). An elicitor‐induced transcription factor WRKY23 was implicated in poplar response to rust infection via its negative regulation of cell wall biogenesis and flavonoid pathway genes (Levee *et al*., 2009). Transcripts of flavonoid biosynthetic genes, including those involved in PA biosynthesis, increased after SA application (Wang *et al*., 2013), indicating a role for SA in regulating these antifungal phenolics. Transcripts of JA biosynthetic genes were upregulated after rust infection of a hybrid poplar (Azaiez *et al*., 2009). However, a recent study reported that SA levels increased in black poplar after rust infection but JA levels did not (Eberl *et al*., 2017), making it unclear whether JA is involved in poplar defense against pathogen attack.

The main objective of this study was to investigate the role of phytohormones in the regulation of flavan‐3‐ol accumulation during poplar–rust interactions. We quantified phytohormone concentrations in black poplar leaves with and without rust fungus inoculation, and found that SA, JA, and ABA all increased in rust‐infected leaves in a time‐dependent manner. Next, we manipulated hormone levels by external spraying, genetic transformation, and/or application of mild drought stress. Both SA and ABA were found to contribute to poplar resistance to rust, by transcriptional activation of flavan‐3‐ol biosynthesis and by reducing stomatal aperture, respectively.

## Materials and Methods

### Plant material and growth conditions

A black poplar genotype (*Populus nigra* L. NP1) obtained from a natural population in northeastern Germany (52°34′1″N, 14°38′3″E) was the principal plant line used in this study. The commercially available hybrid poplar *Populus* × *canadensis* clone Robusta was employed for the drought stress experiment. In addition, wild‐type (WT) and transgenic plants constitutively expressing a bacterial SA synthase with a ferredoxin (FD) plastid‐targeting presequence (*FD‐Irp9*) or SA hydroxylase (*NahG*), for SA overproduction or degradation, respectively, in a *Populus tremula *× *alba* INRA 717‐1B4 background (Xue *et al*., 2013) were used for hormone and phenolics profiling. Young poplar trees were propagated from stem cuttings and grown in the glasshouse under the conditions described by Ullah *et al*. (2017) or Frost *et al*. (2012). Trees with a height of 80–100 cm and between 15 and 20 leaves were used in all experiments unless stated otherwise.

### Inoculation of poplar leaves with rust fungus

Freshly harvested urediniospores of *Melampsora larici‐populina* were used for inoculation experiments. Poplar trees were transferred from the glasshouse to a climate chamber 1 wk before inoculation. A rust spore suspension (*c*. 10^5^ spores ml^−1^ water) was thoroughly sprayed onto the abaxial leaf surface, and mock‐inoculated plants were sprayed with water. Each plant was covered with a polyethylene terephthalate (PET) bag (Bratschlauch, Toppits, Minden, Germany) to maintain high humidity to facilitate spore germination. After 18 h, all bags were opened on top. Leaf samples were collected at different times after inoculation. At each time point, five plants were sampled from both rust‐infected and control treatments. Six leaves from leaf plastochron index (LPI) 5–10 on each tree were harvested, mid‐ribs were removed, and leaf laminae were pooled and immediately frozen in liquid nitrogen. Unless stated otherwise, the same inoculation and sampling techniques were applied in all rust infection experiments. LPI‐5 was harvested from transgenic *P. tremula *×* alba* saplings using similar procedures.

### Extraction of phytohormones and phenolics

Flash‐frozen poplar leaves were ground to a fine powder under liquid nitrogen and then lyophilized. Approximately 10 mg of each lyophilized sample was extracted with 1 ml methanol containing 4 μl phytohormone standard mix (40 ng of D_4_‐SA (Sigma‐Aldrich), 40 ng of D_6_‐JA (HPC Standards GmbH, Cunnersdorf, Germany), 40 ng of D_6_‐ABA (Santa Cruz Biotechnology, Dallas, TX, USA), 8 ng D_6_‐JA‐isoleucine (Ile) conjugate (HPC Standards GmbH)), 5 μg apigenin 7‐glucoside (Sigma‐Aldrich), and 0.4 mg phenyl β‐d *‐*glucopyranoside (Sigma‐Aldrich) as internal standards. The contents were vortexed vigorously for a few seconds, incubated for 25 min at 20°C, and agitated at 1500 rpm, then centrifuged at 13 000 ***g*** at 4°C for 5 min. Approximately 900 μl of the supernatant was transferred to new microcentrifuge tubes. The samples were directly analyzed for phytohormones and phenolics, such as flavan‐3‐ols, other flavonoids, phenolic acids, and salicinoids, by LC–MS. PA oligomers were extracted from *c*. 50 mg freeze‐dried leaf tissue using the method described by Ullah *et al*. (2017).

### Quantification of phytohormones by LC–MS/MS

Phytohormone analysis was performed on an Agilent 1260 high‐performance liquid chromatography (HPLC) system (Agilent Technologies, Santa Clara, CA, USA) attached to an API 5000 tandem mass spectrometer (AB SCIEX, Darmstadt, Germany) equipped with a turbospray ion source operated in the negative ionization mode. Phytohormones were separated on a Zorbax Eclipse XDB‐C18 column (50 mm × 4.6 mm, 18 μm Agilent) at 25°C, with two mobile phases consisting of 0.05% formic acid in (A) water and (B) acetonitrile, at a flow rate of 1.1 ml min^−1^ using the elution profile described by Vadassery *et al*. (2012). The parent ion and corresponding fragments of SA, jasmonates, and ABA were analyzed by multiple reaction monitoring as described earlier (Vadassery *et al*., 2012; Sanchez‐Arcos *et al*., 2016). The concentrations of SA, ABA, JA, and JA‐Ile were determined relative to the corresponding internal standard. The concentration of SAG was determined relative to D_4_‐SA applying a theoretical response factor of 1.0. The concentrations of OH‐JA, JA‐glucoside (Glc) and 12‐sulfo‐JA were determined relative to D_6_‐JA applying a theoretical response factor of 1.0. The levels of 12‐hydroxy‐JA‐Ile (OH‐JA‐Ile) and 12‐carboxy‐JA‐Ile (COOH‐JA‐Ile) were determined relative to the isotopically labeled JA‐Ile standard applying a theoretical response factor of 1.0.

### Quantification of phenolics by LC–MS/MS and HPLC

Flavan‐3‐ol monomers and dimers were analyzed with an Agilent 1200 HPLC system (Agilent Technologies) attached to an API 3200 tandem mass spectrometer (Applied Biosystems, Darmstadt, Germany) and equipped with a turbospray ion source operating in the negative ionization mode. Separation was achieved on a Zorbax Eclipse XDB‐C18 column (50 mm × 4.6 mm, 1.8 μm, Agilent). Formic acid (0.05%) in water and acetonitrile was employed as mobile phases A and B, respectively. The elution profile and other parameters were the same as described by Ullah *et al*. (2017). Phenolic acids were analyzed by the same LC–MS/MS system. Acetic acid (0.1%) in water was used as mobile phase A. Salicinoids and rutin were analyzed following the methods described by Boeckler *et al*. (2013). PA oligomers were analyzed by HPLC with fluorescence detection as described by Hammerbacher *et al*. (2014).

### RNA isolation, complementary DNA synthesis, and quantitative reverse transcription PCR

Total RNA from ground leaf tissue was extracted using the Invitrap Spin Plant RNA Mini Kit (Stratec Biomedical, Birkenfeld, Germany) following the manufacturer's instructions and as described by Ullah *et al*. (2017). Reverse transcription of 1 μg RNA into complementary DNA (cDNA) was performed by using SuperScript II reverse transcriptase (Invitrogen) and 50 pmol Oligo(dT)12–18 Primer (Invitrogen) in a reaction volume of 20 μl. The quantitative PCR reactions were performed as described by Ullah *et al*. (2017), using cDNA equivalent to 10–12 ng of total RNA. Transcript abundance was normalized to the abundance of *Ubiquitin* and was calculated from five biological replicates, with two or three technical replicates per biological sample. Relative expression level was determined using the formula $2^{{-\Delta C}_{T}}$. Primer sequences for all genes used in this study are given in Supporting Information Table S1.

### Gene expression analysis of transgenic plants with altered SA levels

Transgenic *P. tremula *× *alba* (INRA 717‐1B4) lines and plant growth conditions were described previously (Xue *et al*., 2013). Total RNAs extracted using the Plant RNA Reagent (Invitrogen) and the Direct‐zol RNA kit (Zymo Research, Irvine, CA, USA) were used for Illumina RNA sequencing (RNA‐Seq) at the US Department of Energy Joint Genome Institute (DOE JGI), as part of the Community Science Program and project led by Robert Schmitz, Chung‐Jui Tsai, and Jeremy Schmutz. The NCBI Sequence Read Archive (SRA) accession numbers for RNA‐Seq data are: SRP146354, SRP146332, and SRP146333 for WT from 27°C, SRP146323, SRP146337, and SRP146350 for WT from 35°C, SRP146326 and SRP146339 for transgenic line F10 from 27°C, and SRP146340, SRP146325, and SRP146322 for F10 from 35°C. Sequence reads were quality checked and mapped onto a variant‐substituted *P. tremula *× *alba* genome (sPta717) v1.1 as described (Xue *et al*., 2015) using Tophat2 v2.0.12 (Kim *et al*., 2013). Transcript abundance was estimated by HTSeq (Anders *et al*., 2015) and differential expression performed using DESeq2 (Love *et al*., 2014). Microarray expression data were extracted from a previous study (Xue *et al*., 2013).

### Quantification of rust fungus by quantitative reverse transcription PCR and histochemical staining

The transcript levels of the *M. larici‐populina Actin* gene were determined by quantitative reverse transcription (qRT) PCR and normalized to poplar *Ubiquitin* mRNA levels to calculate relative rust growth. For microscopy, leaves were harvested at 7 d post inoculation (dpi) to observe fungal colonization. Leaf discs from the infected leaves were cleared by placing them immediately into boiling 70% (v/v) ethanol for 10 min in a water bath. After subsequent renewal of the ethanol solution, a few drops of lactophenol cotton blue were added, and discs were cleared by placing them in saturated chloral hydrate (25%) for 2 d. The leaf discs were mounted in 60% glycerol and observed under a stereomicroscope (Stemi 2000‐CS, Carl Zeiss Microscopy GmbH, Jena, Germany) and an inverted light microscope (Axiovert 200, Carl Zeiss).

### Exogenous spraying of chemicals before rust inoculation

The following chemicals were purchased to manipulate the hormone concentrations in poplar trees: SA analog BTH (Sigma‐Aldrich, Germany), methyl jasmonate (MeJA; Sigma‐Aldrich), and (+/−)‐ABA (Sigma‐Aldrich). BTH (200 μM and 1 mM), MeJA (100 μM), and ABA (100 μM) were dissolved in 0.2% methanol. The solutions were generously sprayed onto both surfaces of black poplar leaves until liquid dripped off the leaves. Mock‐treated plants were sprayed only with 0.2% methanol in water. After spraying, the plants were covered with PET bags. All PET bags were removed after 18 h. Then after an additional 6 h, four plants from each treatment were sampled (0 dpi) and another 10 plants from each group were inoculated with rust fungus. Samples were collected from five trees per treatment at 3 and 7 dpi as described earlier (see Fig. 4a). The setup of the second spraying experiment using only BTH (1 mM) was similar, except corresponding uninfected control trees were included to compare with rust‐inoculated trees.

### Drought stress treatments followed by rust inoculation

Thirty hybrid poplar *P. *×* canadensis* (clone Robusta) plants of similar height (*c*. 80 cm) having approximately equal numbers of leaves (30) were chosen for this experiment. Fifteen trees were watered normally (*c*. 100 g per plant per day) at 13:00 h and 15 plants were exposed to a mild drought stress (*c*. 50 g water per plant per day). Soil moisture levels corresponding to 80% and 40% field capacity were considered normal and mild drought stress, respectively (Jia *et al*., 2016). After 7 d, five trees from each group were sampled and the remaining 10 plants per treatment were inoculated with *M. larici‐populina*. The watering and drought‐stress treatments were continued until further sampling at 4 and 8 dpi (see Fig. 8a).

### Stomatal aperture measurements

Stomatal aperture size was measured *in planta* according to the method described by Wu & Zhao (2017). Stomatal aperture was monitored in plants sampled at 4 dpi using LPI 4. Transparent nail polish was painted on the abaxial leaf surface and allowed to dry for 5 min. Transparent sticky tape was used to seal the edges of the nail polish. The sticky tape was peeled off along with the dried nail polish and mounted onto a clean glass slide. Two slides were prepared from each leaf (both sides of the midrib), and three microscopic fields per slide were photographed using an inverted light microscope (Axiovert 200) coupled with a camera (AxioVision). Stomatal apertures (> 100 stomata per leaf section) were measured at their mid‐point using ImageJ software (https://imagej.nih.gov/ij/index.html).

### Statistical analysis

All data were analyzed using R v.3.2.0. Normality of data and homogeneity of variances were determined using the Shapiro–Wilk and the Levene test respectively. If testing assumptions were not met, data were square‐root or log transformed. Data were then analyzed by parametric tests such as ANOVA and Student's *t*‐test. Salicylates and flavan‐3‐ol levels in rust‐infected and mock‐treated plants at each time point, were compared using a two‐tailed Student's *t*‐test. Data on defense hormones and expression of signaling genes over the course of infection were analyzed by two‐way ANOVA with two independent variables ‘treatment (rust vs control)’ and ‘time points’. All data obtained after exogenous or genetic manipulations of hormones were analyzed by one‐way ANOVA followed by Tukey's post‐hoc test at 95% confidence. A Student's *t*‐test was used to compare between the two means of the drought‐stress experiment at each time point.

## Results

### Rust infection stimulates foliar levels of flavan‐3‐ols and major defense hormones in black poplar

**Figure 1:**
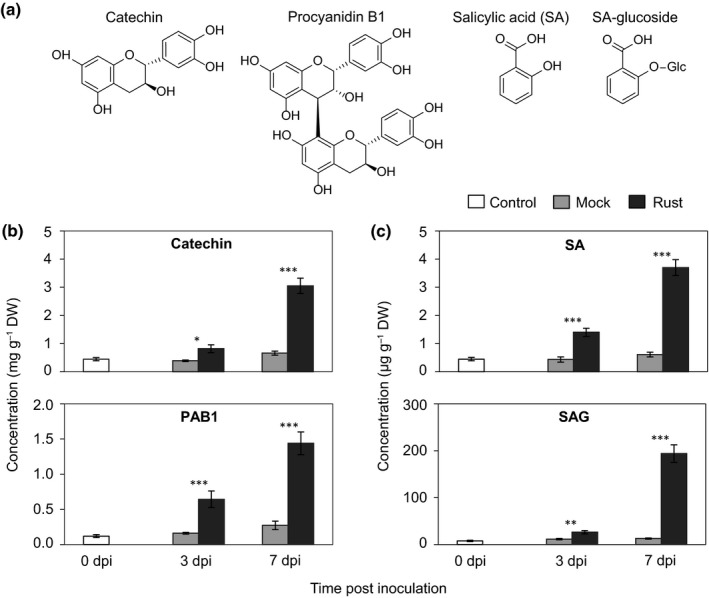
Flavan‐3‐ols and salicylates accumulate to increasing amounts in rust‐infected black poplar leaves. (a) Structures of the proanthocyanidin monomer catechin (2,3‐*trans*‐(+)‐flavan‐3‐ol), procyanidin dimer 1 (PAB1), salicylic acid (SA) and salicylic acid glucoside (SAG). (b) Concentration of catechin and PAB1 in poplar leaves with or without rust infection at two different time points after inoculation. (c) Concentration of SA and SAG. Data were analyzed by Student's *t*‐test: *, *P *≤* *0.05; **, *P *<* *0.01; ***, *P *<* *0.001. Bars represent the mean ± SE (*n *=* *5, with each replicate consisting of a pool of five fully expanded leaves from a single tree (leaf plastochron index 6–10). dpi, days post inoculation.

Catechin (2,3‐*trans*‐(+)‐flavan‐3‐ol, Fig. 1a) levels increased steadily and significantly in rust‐infected *P. nigra* leaves at 3 and 7 dpi relative to mock‐treated leaves (Fig. 1b). The flavan‐3‐ol dimer procyanidin B1 (PAB1, Fig. 1a) also increased correspondingly over the course of infection (Fig. 1b). The defense hormone SA and its glucose‐conjugate SAG both increased significantly, by up to eight‐ and 18‐fold, respectively in rust‐infected leaves compared with mock‐inoculated control (Fig. 1c). These data indicate that the accumulation of flavan‐3‐ols and SA is co‐regulated in rust‐infected black poplar leaves.

**Figure 2:**
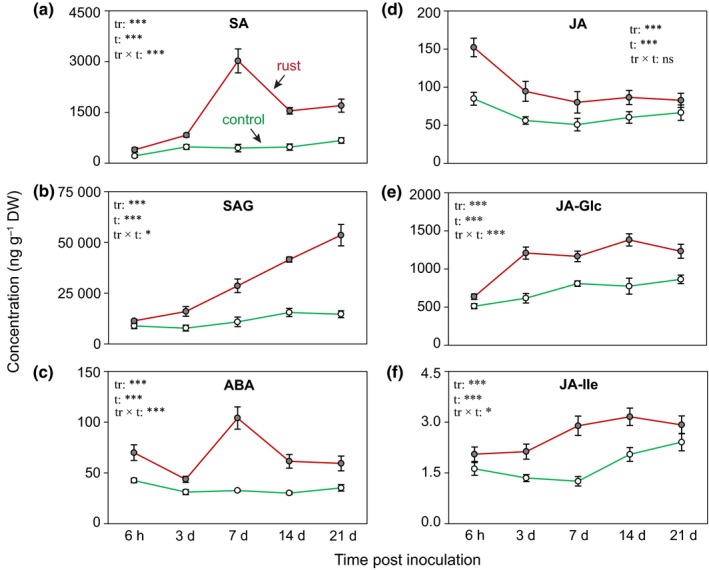
Levels of phytohormones increase in *Populus nigra* leaves over the course of rust fungus infection. Hormone metabolites were analyzed in leaves of rust infected (closed circles) and noninfected control (open circles) trees at different time points after inoculation. Samples were collected from separate trees at each time point. Concentration of (a) salicylic acid (SA), (b) salicylic acid glucoside (SAG), (c) abscisic acid (ABA), (d) jasmonic acid (JA), (e) jasmonic acid glucoside (JA‐Glc), and (f) jasmonic acid‐isoleucine (JA‐Ile). Data were analyzed by two‐way ANOVA. Data are presented as the mean ± SE (*n *=* *5), with each replicate consisting of a pool of six fully expanded leaves (leaf plastochron index 5–10). tr, treatment; t, time post inoculation; ns, nonsignificant; *, *P *≤* *0.05; ***, *P *≤* *0.001.

To investigate changes in hormone concentrations upon rust inoculation more precisely, a kinetic infection experiment was conducted. Black poplar leaves were inoculated with *M. larici‐populina* or treated with water (control), and phytohormone levels were monitored for up to 21 dpi. SA concentrations significantly increased in rust‐infected leaves and peaked at 7 dpi (Fig. 2a). A steady accumulation of the more abundant SAG was observed in rust‐infected plants over the course of infection (Fig. 2b). ABA levels were also induced by rust infection, with the highest concentrations at 7 dpi (Fig. 2c). JA increased rapidly after inoculation, but the response diminished over time (Fig. 2d). By contrast, the biosynthetic precursor of JA, *cis*‐OPDA, did not change in response to infection (Fig. S1). However, the JA catabolites, JA‐glucoside (JA‐Glc) and 12‐sulfojasmonic acid, increased significantly over the course of infection, as did the level of the JA‐Ile and its catabolites, OH‐JA‐Ile and COOH‐JA‐Ile (Figs 1e,f, S1).

We also compared hormone and flavan‐3‐ol levels in young, rust‐free leaves of both rust‐infected and mock‐inoculated plants. The concentrations of flavan‐3‐ols, SA, SAG, and ABA significantly increased in systemic leaves of rust‐infected plants at 7 dpi, but JA levels did not change (Fig. S2). Taken together, these data show that the major defense hormones were significantly upregulated in rust‐infected poplar leaves either at a specific time point or more generally over the course of infection. However, it is unclear which signals specifically trigger the antifungal defense reactions during this compatible interaction.

### Black poplar activates defense signaling genes in response to rust infection

**Figure 3:**
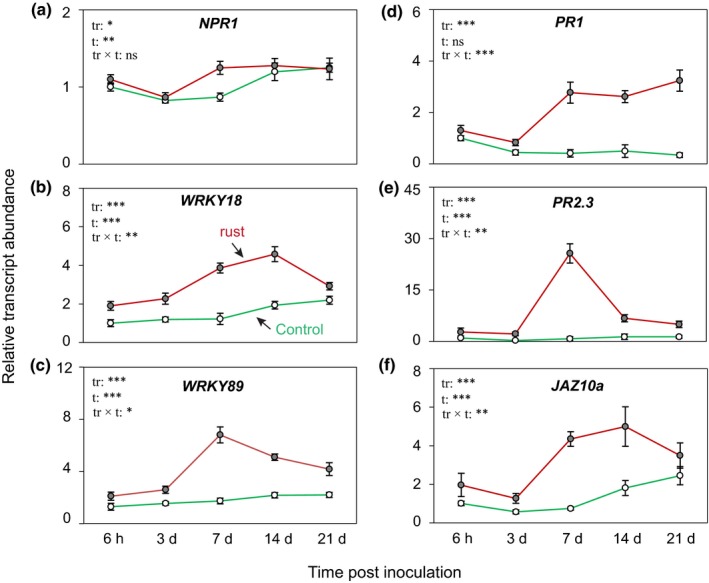
Activation of salicylic acid and jasmonic acid defense signaling genes in rust‐infected leaves of *Populus nigra* trees. Samples were collected from separate trees at each time point. The messenger RNA levels of (a, b) *WRKY* transcription factors, (c) nonexpresser of pathogenesis‐related gene (*NPR1*), (d, e) pathogenesis‐related (*PR*) genes, and (f) jasmonate ZIM‐domain encoding gene (*JAZ10a*) were measured by quantitative reverse transcription PCR in three technical replicates per sample. Transcript levels of each gene were normalized to *Ubiquitin* expression. Corresponding hormone data are depicted in Fig. 2. Data were analyzed using a two‐way ANOVA. Data are presented as the mean ± SE (*n *=* *5), with each replicate consisting of a pool of six fully expanded leaf laminae from a single tree (leaf plastochron index 5–10). tr, treatment; t, time post inoculation; ns, nonsignificant; *, *P *≤* *0.05; **, *P *≤* *0.01; ***, *P *≤* *0.001.

To determine whether the increases in hormones observed upon rust infection activated downstream signaling pathways, we analyzed the relative expression levels of genes potentially involved in defense signaling using qRT‐PCR. An ortholog of *AtNPR1*, the master regulator of SA signaling in *Arabidopsis* (Cao *et al*., 1997), was expressed at very low levels and slightly increased after rust infection at 7 dpi (Fig. 3a). Transcript levels of the WRKY transcription factors *WRKY18*,* WRKY89* (Fig. 3b,c), and *WRKY70* (Fig. S3), known to be stimulated by SA (Jiang *et al*., 2014), increased significantly in rust‐infected leaves, but not *WRKY23* (Fig. S3) previously implicated in poplar–rust interactions (Levee *et al*., 2009). Genes encoding pathogenesis‐related proteins (*PR*s), known SA signaling markers, had been shown to be induced by rust previously (Rinaldi *et al*., 2007; Hamel *et al*., 2011). Here, accumulation of *PR1* transcripts increased threefold (Fig. 3d), and *PR2*.3 transcripts increased up to 30‐fold in rust‐infected leaves (Fig. 3e). However, the relative expression of *PR5* was significantly lower in the foliage of rust‐infected trees than in control plants over the course of infection (Fig. S3). Transcription of the JA signaling gene *JAZ10* significantly increased after rust infection at 7 and 14 dpi compared with the corresponding control plants (Fig. 3f).

### Pretreatments of BTH and ABA enhance black poplar resistance to rust

**Figure 4:**
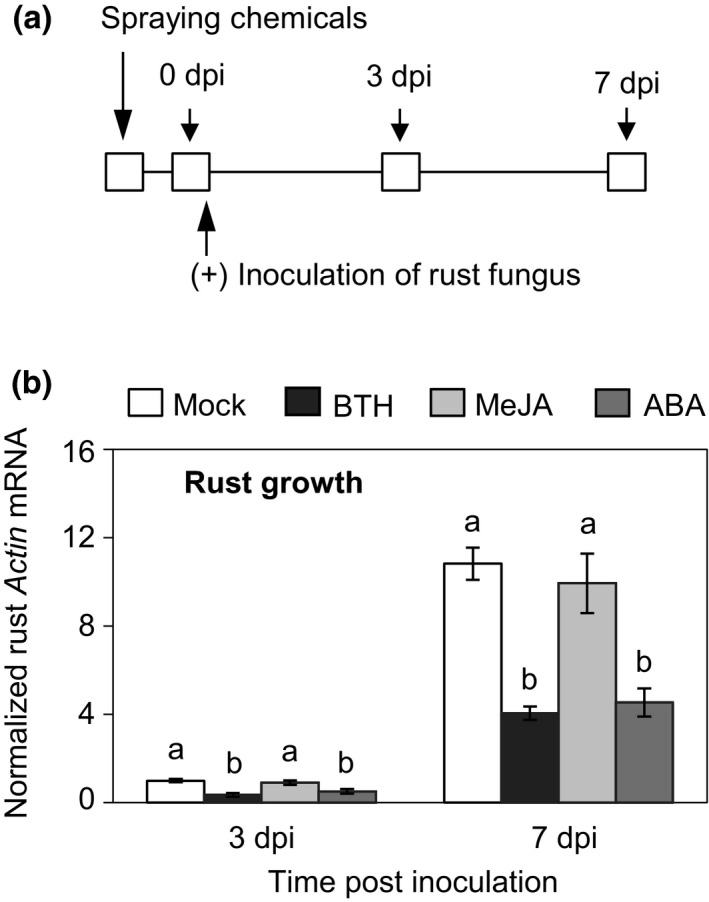
Pretreatment with benzothiadiazole (BTH) and abscisic acid (ABA) reduces the colonization of rust fungus in the foliage of black poplar. (a) Experimental outline. Young black poplar trees were sprayed with BTH (200 μM in 0.2% methanol), methyl jasmonate (MeJA, 100 μM), ABA (100 μM) or with 0.2% methanol (mock). A subset of plants from each group was sampled 1 d after spraying (0 d post inoculation (dpi) of rust fungus) and the remaining trees were inoculated with rust spores. Leaf laminae were collected from separate trees at 3 and 7 d after rust inoculation. (b) Relative colonization of rust fungus in different poplar trees over the course of infection. *Melampsora larici‐populina actin* messenger RNA levels were normalized to poplar *ubiquitin* messenger RNA levels to calculate relative rust growth. Time‐course data were analyzed using a one‐way ANOVA followed by Tukey's post‐hoc test. Different letters indicate means were statistically different at 95% confidence level. Data are presented as the mean ± SE (*n *=* *5), with each replicate consisting of a pool of six fully expanded leaves (leaf plastochron index 5–10) from a single tree. Three technical replicates were used per sample during quantitative reverse transcription PCR.

**Figure 5:**
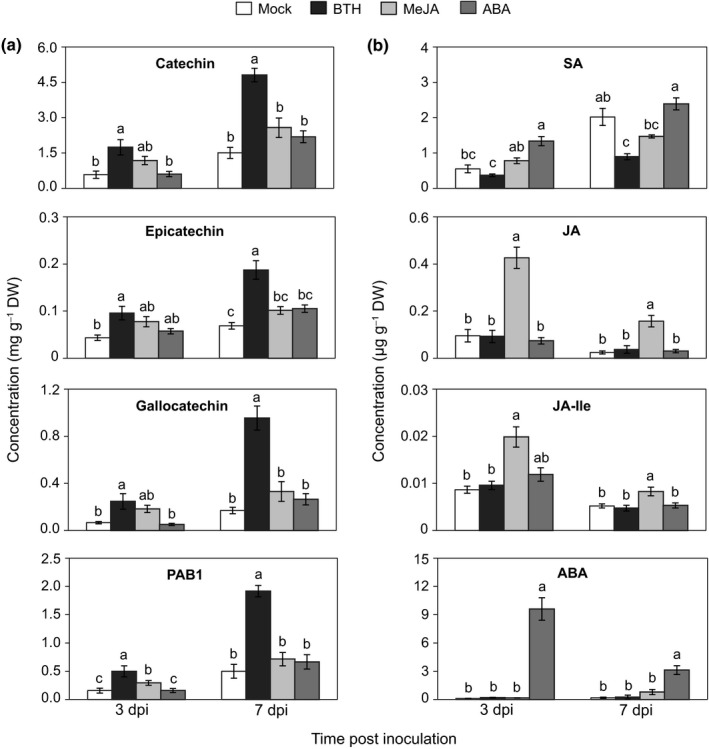
Effect of defense hormone treatment on (a) flavan‐3‐ols and (b) endogenous hormones in *Populus nigra* leaves after rust infection. Poplar trees were treated with hormones and inoculated with fungus 24 h later. Defense hormones and flavan‐3‐ol monomers and dimers were analyzed by LC–MS/MS. Time‐course data were analyzed using a one‐way ANOVA followed by Tukey's post‐hoc test. Different letters indicate groups that were statistically different at 95% confidence level. Data are presented as the mean ± SE (*n *=* *5), with each replicate consisting of a pool of six fully expanded leaf laminae (leaf plastochron index 5–10) from a single tree. ABA, abscisic acid; BTH, benzothiadiazole; JA, jasmonic acid; JA‐Ile, jasmonic acid‐isoleucine; MeJA, methyl jasmonate; PAB1, procyanidin dimer 1; SA, salicylic acid; dpi, days post inoculation of rust fungus.

To test whether exogenous phytohormones could activate poplar defense against rust infection, we treated black poplar saplings with BTH (a functional analog of SA), MeJA, and ABA for 24 h before inoculation with *M. larici‐populina* (Fig. 4a). At 3 dpi, the time when the fungus establishes a hyphal network in mesophyll tissues without visible symptoms, the relative growth of the fungus was significantly lower in BTH‐ and ABA‐treated plants than in mock‐ and MeJA‐treated plants (Fig. 4b) by *c*. 40–50%. A similar rust colonization pattern was observed at 7 dpi, when uredinia appear on the lower surface of the leaves. These samples were then subjected to phenolic profiling. The most striking differences between pretreatments were seen in flavan‐3‐ols. Catechin levels were significantly higher in rust‐infected leaves pretreated with BTH than with ABA, MeJA, or mock treatments (Fig. 5a). Similar patterns were also observed for epicatechin, gallocatechin, and dimeric PAB1 (Fig. 5a). The levels of salicinoids, phenolic acids, and the flavonoid rutin were largely unchanged by the pretreatments except for phenolic acids, which were slightly reduced by BTH (Table S2; Fig. S4).

The MeJA treatment significantly increased the levels of JA (> 25‐fold), JA‐Ile, and other JA conjugates and catabolites in poplar leaves after 1 d (Table S3). Similarly, ABA levels increased by > 1000‐fold 1 d following ABA application (Table S3). After rust infection, the MeJA‐ and ABA‐induced differences diminished over time, but remained significant through 7 dpi (Figs 5b, S5). BTH‐treated poplars contained slightly lower levels of SA than the mock‐treated plants did (Table S3). Similar patterns persisted after rust inoculation, with the difference becoming significant at 7 dpi (Fig. 5b).

### BTH stimulates flavan‐3‐ol biosynthesis independent of rust infection

**Figure 6:**
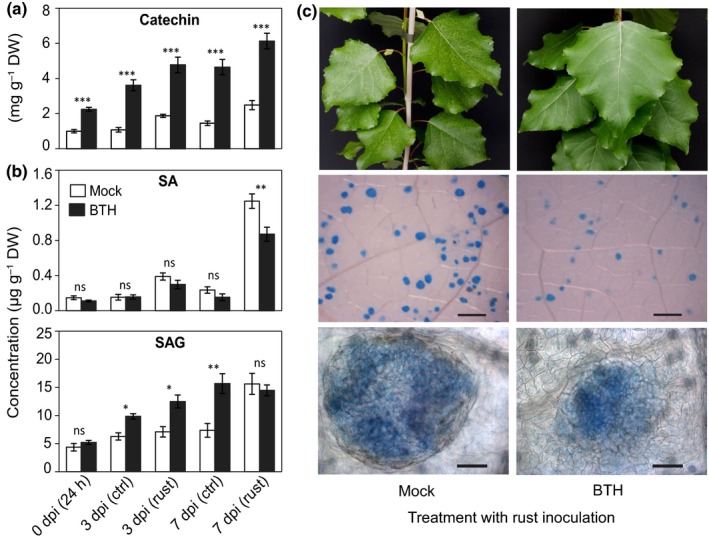
Benzothiadiazole (BTH) treatment increases rust resistance in black poplar and increases flavan‐3‐ol accumulation with or without rust infection. Black poplars were sprayed with BTH (1 mM in 0.2% methanol) and corresponding control plants were treated with 0.2% methanol. One day after spraying, a subset of plants from each treatment was sampled and the remaining plants were inoculated with either rust spores or with water as a control. Samples were collected from separate trees 3 and 7 d post inoculation (dpi). Depicted are the concentrations of (a) catechin, (b) salicylic acid (SA) and SA‐glucoside (SAG), and (c) the colonization of rust fungus in poplar leaves 7 dpi (top panels, rust‐infected leaves; middle panels, fungus stained with lactophenol blue after clearing the leaves; bottom panels, single uredinia after staining). Bars: middle panels, 500 μm; bottom panel, 20 μm. Data (mock vs BTH) were analyzed using a two‐tailed Student's *t*‐test: ns, nonsignificant; *, *P *≤* *0.05; **, *P *<* *0.01; ***, *P *<* *0.001. Data are presented as the mean ± SE (*n *=* *4), and each replicate was a pool of six fully expanded leaves (leaf plastochron index 5–10) from a single tree. ctrl, water‐treated control; rust, rust inoculated.

To clarify whether BTH‐triggered flavan‐3‐ol accumulation occurs exclusively under pathogen attack or also without rust inoculation, we treated poplar plants with a higher concentration of BTH (1 mM) and compared the accumulation of flavan‐3‐ol metabolites with or without rust infection. Already 24 h after BTH treatment but before rust inoculation (0 dpi), catechin accumulation was significantly higher in BTH‐treated than in mock‐treated plants (Fig. 6a). Similar patterns were also observed in trees at 3 and 7 dpi regardless of rust infection (Fig. 6a). The levels of gallocatechin and PA dimers were also higher in BTH‐treated plants, both with and without rust infection (Fig. S6). The levels of SA slightly decreased in BTH‐treated plants, but significant differences were only observed after rust infection at 7 dpi (Fig. 6b). Interestingly, concentrations of the abundant SAG were significantly higher in BTH‐treated plants throughout the monitoring period regardless of rust infection (Fig. 6b). The level of exogenous BTH gradually declined over time but was still detectable at 7 dpi (Fig. S6). As expected, rust colonization, as well as sporulation, was reduced in poplar trees that were pretreated with BTH (Fig. 6c). Taken together, these results suggest that the SA analog BTH increased black poplar resistance against foliar rust infection by triggering flavan‐3‐ol accumulation.

**Figure 7:**
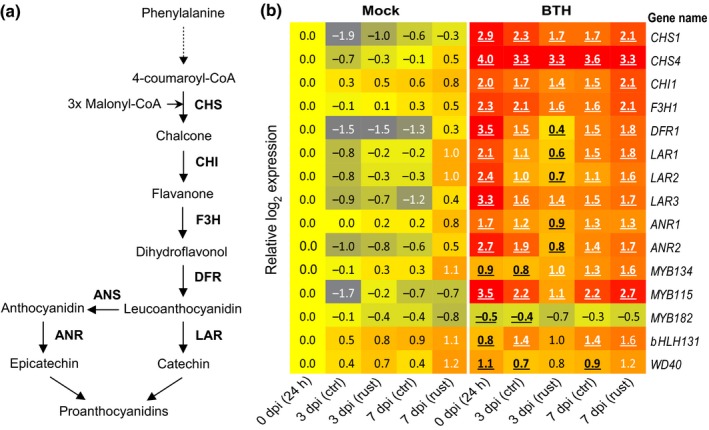
Transcriptional upregulation of genes encoding transcription factors and enzymes involved in the biosynthesis of proanthocyanidins (PAs) in black poplar treated with benzothiadiazole (BTH). (a) Flavonoid pathway leading to the formation of catechin and PAs. (b) Relative expression of PA biosynthesis genes and MYB transcription factors regulating this pathway. Transcript levels were measured by quantitative reverse transcription PCR using two technical replicates per sample. Transcripts of each gene were normalized to *ubiquitin* expression. The heat map was generated using the average values (log_2_‐transformed) of five biological samples per treatment. Time‐point data (mock vs BTH) were analyzed using a Student's *t*‐test. Bold underlined values are significantly different between BTH‐ and mock‐treated samples (*P *≤* *0.05). CHS, chalcone synthase; CHI, chalcone isomerase; F3H, flavanone 3‐hydroxylase; DFR, dihydroflavonol reductase; FLS, flavonol synthase; LAR, leucoanthocyanidin reductase; ANS, anthocyanidin synthase; ANR, anthocyanidin reductase. Note that MYB134 and MYB115 positively regulate PA synthesis in association with the basic helix–loop–helix enzyme bHLH131 (James *et al*., 2017). MYB182 acts as a negative regulator of PA synthesis in poplar (Yoshida *et al*., 2015).

To reveal the mechanism of BTH‐stimulated flavan‐3‐ol accumulation in poplar, we analyzed the expression of flavonoid pathway genes leading to PA synthesis by qRT‐PCR (Fig. 7a). Our data (Fig 7b) clearly demonstrated that transcript levels of chalcone synthase (*CHS1*,* CHS4*), chalcone isomerase (*CHI1*), flavanone 3‐hydroxylase (*F3H1*), dihydroflavonol reductase (*DFR1*), leucoanthocyanidin reductase (*LAR*), and anthocyanidin reductase (*ANR*) increased significantly in BTH‐treated plants compared with the corresponding mock‐treated trees. Moreover, the transcriptional regulators (*MYB134*,* MYB115*,* bHLH*, WD40) of PA biosynthesis were also upregulated upon BTH treatment, whereas the negative regulator (*MYB182*) was slightly lower in BTH‐treated saplings (Fig. 7b).

### Endogenously elevated SA promotes flavan‐3‐ol biosynthesis and accumulation

**Figure 8:**
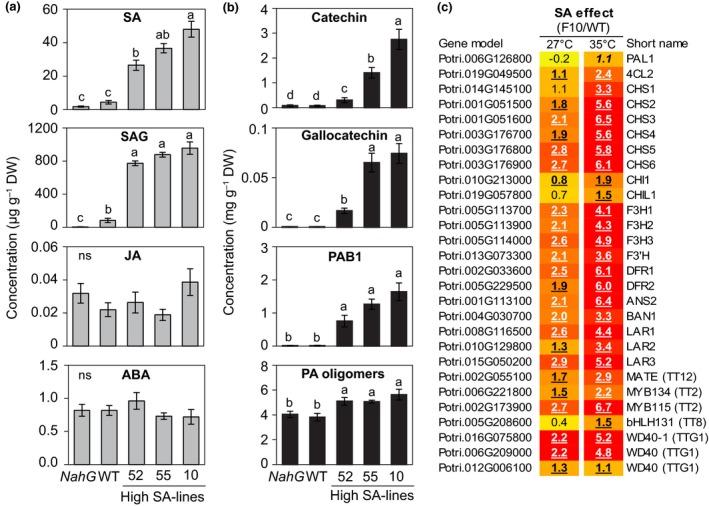
Transgenic poplar (*Populus tremula* × *alba*) overproducing salicylic acid (SA) accumulates higher levels of flavan‐3‐ols. SA overproduction is due to expression of a bacterial synthase gene (*Irp9*) with a ferredoxin (*FD*) plastid‐targeting sequence. Depicted are the concentrations of (a) defense hormones and (b) flavan‐3‐ol monomers (catechin and gallocatechin), the principal flavan‐3‐ol dimer (procyanidin B1, PAB1), and flavan‐3‐ol oligomers (PA oligomers) measured up to 10 monomeric units. PA oligomers were calculated as catechin equivalents. Leaf samples (leaf plastochron index 5) were collected from 6‐wk‐old poplar trees. Data were analyzed using a one‐way ANOVA followed by Tukey's post‐hoc test, and different letters indicate that groups were statistically different (*P *<* *0.05). Data are presented as the mean ± SE (*n *=* *3). SAG, SA glucoside; ABA, abscisic acid; JA, jasmonic acid; WT, wild‐type; *NahG*, gene encoding SA hydroxylase; ns, nonsignificant. (c) Heat map depiction of the effects of SA on PA gene expression quantified by RNA sequencing. Transcript abundances of WT and high‐SA‐producing plants at two different growth temperatures were converted to expression ratios, log_2_‐transformed and visualized in the heat map. Yellow indicates no change in expression; red denotes upregulation. Statistical significance is denoted by bold underlined (*P *<* *0.01) or bold italics (*P *<* *0.05), with *n *=* *3 biological replicates, except F10 at 27°C (*n *=* *2).

The aforementioned data strongly suggest SA as a central player in PA‐mediated rust resistance in black poplar. To more directly assess the role of SA in PA biosynthesis, we explored a pathogen‐free system using three transgenic *P. tremula* × *alba FD‐Irp9* lines with constitutively elevated levels of SA and SAG (Xue *et al*., 2013). For comparison, we included one WT and an *NahG* line (expressing a bacterial SA hydroxylase) for flavan‐3‐ol analysis. As expected, the levels of SA and SAG were significantly higher in the *FD*‐*Irp9* plants than in WT plants, by up to 12‐fold, whereas the levels were lower in *NahG* plants (Fig. 8a). The concentrations of JA and ABA, on the other hand, did not change in SA overproducing lines in comparison with WT and the *NahG* line (Fig. 8a).

Consistent with the results of BTH spraying, SA overproducing plants accumulated significantly higher amounts of flavan‐3‐ol monomers, such as catechin and gallocatechin, as well as dimeric PAB1, than in WT and *NahG* plants (Fig. 8b). Furthermore, PA oligomers (up to 10–12 monomeric units) were 25–30% higher than in WT plants (Fig. 8b). These data strongly support the hypothesis that SA positively regulates flavan‐3‐ol biosynthesis in poplar.

RNA‐Seq analysis confirmed significant upregulation of essentially all known flavan‐3‐ol biosynthetic pathway genes in a high‐SA (F10) line relative to the control (Fig. 8c). Previous work has shown that SA and SAG levels were higher in transgenic plants grown at 35°C than at 27°C (Xue *et al*., 2013). The stronger transcriptional induction of PA biosynthetic genes in transgenic leaves at 35°C than at 27°C (Fig. 8c) is therefore consistent with SA‐dependent regulation of PA biosynthesis. Moreover, transcription factors MYB115, MYB134, bHLH, and WD40 were also transcriptionally co‐regulated with the flavonoid pathway genes (Fig. 8c), suggesting that SA stimulates PA biosynthesis by activating the MBW complex.

### Increased levels of ABA under mild drought conditions reduces rust colonization via stomatal closure without altering flavan‐3‐ol levels

**Figure 9:**
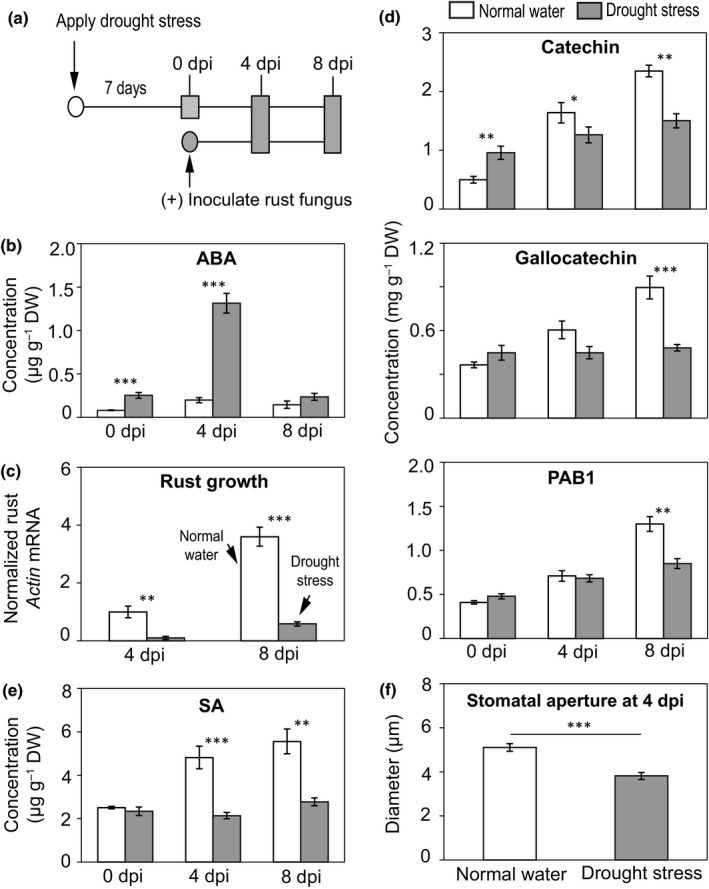
Mild drought stress in poplar (*Populus* × *canadensis* Robusta) increases resistance to rust fungus infection. (a) Experimental outline. Poplar saplings were watered normally or exposed to mild drought conditions. After 7 d, a subset of plants was sampled and the remaining plants were inoculated with *Melampsora larici‐populina*. The drought treatment was continued until the end of the experiment. (b) Concentrations of abscisic acid (ABA). (c) Relative rust colonization under mild drought or normal watering treatment at 3 and 7 d post inoculation (dpi). *M. larici‐populina Actin* messenger RNA levels were normalized to poplar *Ubiquitin* messenger RNA levels to calculate relative rust growth. (d) Concentrations of flavan‐3‐ol monomers catechin and gallocatechin, and the flavan‐3‐ol dimer procyanidin B1 (PAB1) under mild drought and normal watering treatments with or without rust inoculation. (e) Concentrations of salicylic acid (SA). (f) Stomatal aperture at 4 dpi under mild drought or normal watering treatment. Data were analyzed using a two‐tailed Student's *t*‐test: *, *P *≤* *0.05; **, *P *≤* *0.01; ***, *P *≤* *0.001. Data are presented as the mean ± SE (*n *=* *5) and each replicate consisted of a pool of five fully expanded leaves (leaf plastochron index 5–10) from a single tree.

The induction of ABA in black poplar upon infection by the rust fungus (Fig. 2c) and the increased resistance against rust after exogenous ABA treatment (Fig. 4b) suggested that ABA also plays a role during poplar–rust interactions. The biosynthesis of ABA is known to be upregulated in many plants, including poplars, under drought stress (Cutler *et al*., 2010; Jia *et al*., 2016). To investigate whether poplar response to rust infection is altered under drought stress, we subjected poplar saplings to mild drought stress followed by rust inoculation (Fig. 9a). As expected, the endogenous levels of ABA increased approximately threefold after 7 d of mild drought stress (0 dpi), and further increased up to 16‐fold (1.3 μg g^−1^ leaf DW) in response to sustained drought stress and rust infection at 4 dpi, before returning to basal levels at 8 dpi (Fig. 9b). Under mild drought stress, the growth of *M*. *larici‐populina* significantly decreased by 10‐fold and sixfold at 4 dpi and 8 dpi, respectively (Fig. 9c).

We described earlier that short‐term exogenous ABA treatments did not affect flavan‐3‐ol accumulation (Fig. 5; Table S2). Interestingly, catechin levels increased significantly after 7 d of mild drought (0 dpi), although SA levels did not change (Fig. 9d,e). However, drought stress abolished the typical rust‐induced accumulations of SA and PAs (catechin, epicatechin, gallocatechin, and PAB1) at 4 and 8 dpi (Figs 9e, S7). Thus, the drought‐related resistance to rust infection cannot be attributed to elevated SA or flavan‐3‐ol levels. Levels of jasmonates, salicinoids, and phenolic acids in drought‐stressed and rust‐infected leaves did not change (Fig. S7). Analysis of stomatal opening revealed a significantly reduced aperture in drought‐stressed plants after rust inoculation (Fig. 9f). These findings indicate that, under mild drought stress, ABA induces stomatal closure that could reduce entry of the pathogen to confer resistance independent of flavan‐3‐ols.

## Discussion

**Figure 10:**
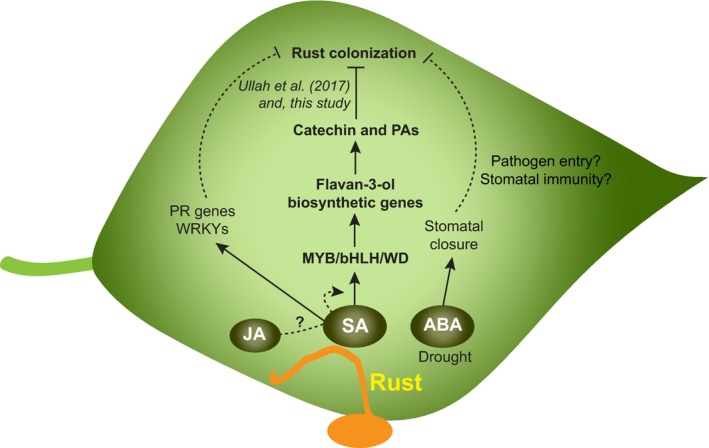
Hormone‐mediated defenses in poplar against rust infection. The biosynthesis of salicylic acid (SA), jasmonic acid (JA), and abscisic acid (ABA) are all upregulated in poplar leaves colonized by the rust fungus *Melampsora larici‐populina*. SA stimulates expression of WRKY transcription factors and pathogenesis‐related (PR) genes. SA induces transcription of MYB134, MYB115, bHLH131, and WD40, which then upregulate a large set of flavan‐3‐ol biosynthetic genes. Increasing flavan‐3‐ol biosynthesis in turn leads to accumulation of catechin and PAs, which negatively influence rust colonization. ABA also increases poplar resistance by inhibiting pathogen entry via stomatal closure. Solid lines indicate findings supported by experimental evidence obtained in this study.

In nature, poplar trees are constantly challenged by a plethora of pathogens. Among these, rust fungi (*Melampsora* spp.) are the most widespread and destructive pathogens. These obligate biotrophs do not kill their hosts, but decrease biomass production by reducing photosynthesis and stimulating early defoliation (Newcombe *et al*., 2001; Duplessis *et al*., 2009). To limit rust severity, poplar synthesizes high amounts of flavan‐3‐ols, including monomeric catechin and polymeric PAs (Ullah *et al*., 2017). Here, we investigated the roles of hormones in poplar defense against rust infection with particular emphasis on the regulation of flavan‐3‐ol biosynthesis. The levels of SA, JA, and ABA all increased in black poplar leaves during rust infection. However, local rust infection resulted in increased concentrations of only SA and ABA in systemic leaves without changing the levels of JA. Poplar trees that were pretreated with the SA analog BTH or with ABA were less susceptible to rust infection than mock‐ and MeJA‐treated plants were. We show that SA and BTH enhanced rust resistance by increasing catechin and PA accumulation via transcriptional activation of PA biosynthesis in poplars. ABA, on the other hand, increased poplar resistance to rust infection by stimulating stomatal closure (Fig. 10).

### Rust infection induces increases of SA, JA, and ABA in black poplar

SA, JA, and ABA levels increased in rust‐colonized black poplar leaves, but with different temporal responses. In our study, JA accumulated to higher levels during the early colonization phase, whereas SA and ABA reached maximum levels during sporulation with a sustained accumulation of SAG over the course of infection. Rust infection also increased transcript levels of the SA‐ and JA‐responsive *WRKY18* (Jiang *et al*., 2014), the SA‐responsive *WRKY89* (Jiang *et al*., 2016), several *PR* genes (Boyle *et al*., 2010), and the JA‐responsive *JAZ10* (Hamel *et al*., 2011). Together, these data indicate that the JA and SA signaling pathways were both upregulated during rust infection and their antagonism is not evident in poplar–rust interactions as is shown for herbaceous plants (Spoel *et al*., 2003; Pieterse *et al*., 2012). A lack of SA–JA antagonism in poplars was also observed previously. Increased levels of defense‐related gene expression were elicited by both SA and JA applications in an ozone‐tolerant hybrid poplar clone (Koch *et al*., 2000), whereas increased levels of SA, JA, and ABA were detected in young black poplar trees upon herbivore feeding (Clavijo McCormick *et al*., 2014). Likewise, detached leaves of *in vitro Populus davidiana* treated with MeJA showed increased levels of SA (Park *et al*., 2017). However, we observed only a slight increase in SA contents upon MeJA treatment *in planta*, and JA content did not change in transgenic poplars overproducing SA. Thus, it appears that SA and JA are not necessarily antagonistic in poplar.

### SA increases poplar defenses against rust by triggering accumulation of flavan‐3‐ols

Flavan‐3‐ols have been shown as constitutive and induced chemical defenses in poplar against fungal pathogens (Ullah *et al*., 2017; Wang *et al*., 2017b). In this investigation, we show that flavan‐3‐ols increased only after treatments with the SA analog BTH, but not with ABA or MeJA. BTH is well known for its efficacy in activating SA‐mediated defense signaling in a wide range of plant species (Friedrich *et al*., 1996; Lawton *et al*., 1996). In the model system *Arabidopsis*, where SA defense mechanisms have been most extensively studied, SA and BTH are not known to stimulate the flavonoid biosynthetic pathway. However, exogenous SA application to the culture medium of *Cistus heterophyllus* resulted in increased accumulation of PAs in growing shoots (López‐Orenes *et al*., 2013). SA also activates flavonoid biosynthesis and increased PA accumulation in cell suspension cultures of grapevine (Wang *et al*., 2017a). These observations, along with our observations of elevated flavan‐3‐ol accumulation following leaf rust infection and upon protective BTH treatments, implicate SA as the defense hormone mediating PA‐based resistance to rust fungi in poplar.

Analysis of transgenic poplars with constitutively elevated SA (Xue *et al*., 2013) provides independent evidence for a role of SA in PA biosynthesis. In this case, SA hyperaccumulation was achieved by manipulating SA biosynthesis, unlike in many *Arabidopsis* mutants that exhibit increased SA accumulation as a pleiotropic effect due to mutations in defense signaling or other cellular processes (Mateo *et al*., 2006). These transgenic poplars thus permit a direct assessment of the hypothesized role of SA without other confounding factors. We show that these plants constitutively accumulated higher amounts of flavan‐3‐ols than WT and *NahG* plants did. Transgenic SA manipulations also affected other secondary metabolites in poplar (Morse *et al*., 2007; Xue *et al*., 2013), such as reductions of salicinoids in SA‐overproducing poplars. We indeed observed a slight decrease in the levels of salicinoids in BTH‐treated plants, which is likely due to a trade‐off between PA and salicinoid biosynthesis in poplar (Mellway *et al*., 2009; Boeckler *et al*., 2014). These data indicate that SA triggers flavan‐3‐ol biosynthesis only, whereas levels of other abundant phenolic compounds in poplar are either unaffected or reduced due to a metabolic trade‐off.

### SA activates MYB transcription factors to induce flavan‐3‐ol biosynthesis

Gene expression analysis revealed widespread transcriptional upregulation of flavonoid pathway genes involved in PA synthesis by exogenous BTH treatment as well as by endogenous SA increases in poplars. The flavonoid biosynthetic pathway is known to be transcriptionally regulated by the MBW complex (Hichri *et al*., 2011). MYB134 was the first transcription factor characterized in poplar that positively regulates PA biosynthesis (Mellway *et al*., 2009). Recently, MYB115 was shown to be another positive regulator of PA synthesis (James *et al*., 2017; Wang *et al*., 2017b). These two transcription factors work in association with a bHLH131 transcription factor and a WD40 protein in the poplar MBW complex (James *et al*., 2017). We showed that transcript levels of these regulatory genes increased in both BTH‐treated and SA‐hyperaccumulating lines. Therefore, the SA‐mediated flavan‐3‐ol increases in poplar in response to rust infection can be attributed to the transcriptional upregulation of *MYB/bHLH/WD40* genes, which then induce transcription of genes encoding enzymes of the PA biosynthetic pathway. Although the regulatory elements responsible for SA activation of the MBW complex remain to be discovered, this work provides convincing evidence for a direct role of SA in regulating PA biosynthesis in poplar.

### ABA increases poplar defenses against rust by stomatal closure

ABA did not affect SA and flavan‐3‐ol accumulations, although its application improved poplar resistance to subsequent fungus infection. This suggests that ABA‐mediated defense acts independently of SA signaling. Indeed, ABA levels increased in poplar leaves under mild drought stress, and they were further augmented by rust infection at 4 dpi, whereas SA levels did not change during drought and were not responsive to rust infection in drought‐stressed plants. As a result, ABA levels increased but SA levels decreased at 4 dpi when rust growth was reduced. Based on reduced stomatal aperture in drought‐stressed and rust‐infected leaves at 4 dpi, we reason that ABA‐mediated rust resistance is likely associated with stomatal immunity. Stomata are the entry points of many pathogens (Gudesblat *et al*., 2009), and closure of stomata is a defense strategy employed by plants to limit entry of pathogens into leaves (Melotto *et al*., 2006; Sun *et al*., 2014). After germination of *Melampsora* spores on the abaxial surface of poplar leaves, the germ tubes penetrate via stomata (Newcombe *et al*., 2001; Hacquard *et al*., 2011). ABA increased significantly 6 h after infection and reached a maximum level at 7 dpi. Thus, ABA likely acts as an early defense by inducing stomatal closure to prevent entry of rust fungi, and the sustained increases of ABA upon rust infection could strengthen this physical defense during the course of the infection. Consistent with this idea, increased ABA under mild drought stress is associated with reduced stomatal aperture size, which might have limited pathogen entry. Continued increases of ABA resulted in substantially reduced rust colonization throughout our monitoring period, up to 8 dpi, in drought‐stressed plants. A negative association between stomatal density/pore size and rust resistance was reported in poplar using a genome‐wide association study (McKown *et al*., 2014), supporting a role of stomatal immunity in poplar–rust interactions. We suggest that signal transduction during the poplar–rust interaction may be fine‐tuned under drought stress such that increases in ABA confer both drought tolerance and disease resistance by reducing stomatal aperture, and that the SA‐mediated and metabolically costly defense mechanisms are downregulated.

In conclusion, upon infection by the biotrophic rust fungus *M. larici‐populina*, black poplar activates SA, JA, and ABA signaling pathways in its leaves. SA induces the accumulation of catechin and PAs, compounds known as effective defenses against leaf rust infection. ABA also plays a role in rust resistance by triggering reductions in stomatal aperture size that limit fungal entry, a resistance mechanism that may be more important under drought stress with elevated levels of ABA. Thus, the roles of hormones in regulating defense appear to differ between woody perennials and herbaceous plants. We showed no evidence of antagonism between SA and JA in poplar, but many other species should be surveyed before any generalization can be made.

Further study is also necessary to dissect the regulation of stomatal immunity in poplar trees during simultaneous drought and rust infection, two stresses that may be connected by reactive oxygen species (ROS) accumulation. It has been shown that rust infection induces ROS accumulation around the stomata cells in poplar (Boyle *et al*., 2010), and drought stress also induces oxidative stress in poplar leaves (Jia *et al*., 2016). However, increased ROS tolerance in poplar is associated with susceptibility to rust (La Mantia *et al*., 2018). Further questions to be addressed are how rust and drought interact to control ROS accumulation, and whether flavan‐3‐ols themselves are antioxidant or prooxidant under these conditions.

## Author contributions

C.U., S.B.U., J.G., and A.H. conceived and designed the experiments. C.U. performed all experiments and analyzed the data. M.R. assisted in phytohormone analysis. C‐J.T. provided transgenic poplar lines, and analyzed the RNA‐Seq data with help from L.X. C.U. prepared the draft manuscript, which was edited by C‐J.T., S.B.U., J.G., and A.H. All authors read, gave comments, and approved the manuscript.

## Supporting information

Please note: Wiley Blackwell are not responsible for the content or functionality of any Supporting Information supplied by the authors. Any queries (other than missing material) should be directed to the *New Phytologist* Central Office.

**Fig. S1** Effect of rust infection on the concentrations of the jasmonic acid (JA) precursor, *cis*‐OPDA, and JA catabolites in black poplar leaves over the course of infection.
**Fig. S2** Effect of rust infection on the accumulation of flavan‐3‐ols, abscisic acid and salicylic acid in expanding systemic leaves of black poplar trees.
**Fig. S3** Effect of rust infection on the relative expression levels of *WRKY23*,* WRKY70* and *PR5*.
**Fig. S4** Phytohormone application before rust infection did not alter the levels of other phenolic metabolites in poplar besides flavan‐3‐ols.
**Fig. S5** Jasmonate concentrations in *Populus nigra* leaves after exogenous phytohormone application followed by rust infection.
**Fig. S6** Accumulation of flavan‐3‐ols in black poplar leaves treated with the salicylic acid analog benzothiadiazole.
**Fig. S7** Concentrations of jasmonates, epigallocatechin, salicinoids and phenolic acids in poplar (*Populus* × *canadensis* Robusta) leaves under mild drought stress and rust infection.
**Table S1** Primer sequences used in this study.
**Table S2** Levels of phenolic metabolites in black poplar leaves 1 d after exogenous hormone treatment.
**Table S3** Levels of endogenous hormones in black poplar leaves 1 d after exogenous hormone treatment.
