# Supplementary material for: Salicylic acid activates poplar defense against the biotrophic rust fungus Melampsora larici‐populina via increased biosynthesis of catechin and proanthocyanidins
Source: New Phytol. 2018 Aug 31;221(2):960–75. doi: 10.1111/nph.15396 (PMC6585937; doi:10.1111/nph.15396)
Supplement: Supplementary file 1 — Fig. S1 Effect of rust infection on the concentrations of the jasmonic acid (JA) precursor, cis‐OPDA, and JA catabolites in black poplar leaves over the course of infection. Fig. S2 Effect of rust infection on the accumulation of flavan‐3‐ols, abscisic acid and salicylic acid in expanding systemic leaves of black poplar trees. Fig. S3 Effect of rust infection on the relative expression levels of WRKY23, WRKY70 and PR5. Fig. S4 Phytohormone application before rust infection did not alter the levels of other phenolic metabolites in poplar besides flavan‐3‐ols. Fig. S5 Jasmonate concentrations in Populus nigra leaves after exogenous phytohormone application followed by rust infection. Fig. S6 Accumulation of flavan‐3‐ols in black poplar leaves treated with the salicylic acid analog benzothiadiazole. Fig. S7 Concentrations of jasmonates, epigallocatechin, salicinoids and phenolic acids in poplar (Populus × canadensis Robusta) leaves under mild drought stress and rust infection. Table S1 Primer sequences used in this study. Table S2 Levels of phenolic metabolites in black poplar leaves 1 d after exogenous hormone treatment. Table S3 Levels of endogenous hormones in black poplar leaves 1 d after exogenous hormone treatment. [file NPH-221-960-s001.pdf]

Supporting Information

Article title: Salicylic acid activates poplar defense against the biotrophic rust fungus *Melampsora larici-populina via* increased biosynthesis of catechin and proanthocyanidins

Authors: Chhana Ullah, Chung-Jui Tsai, Sybille B. Unsicker, Liangjiao Xue, Michael Reichelt, Jonathan Gershenzon, Almuth Hammerbacher

Article acceptance date: 10 July 2018

The following Supporting Information is available for this article:

Table S1 Primer sequences used in this study

Primer name	Purpose	Primer sequence (5' → 3')	References
WRKY18-for	qPCR	TTATGAAGGAGAGCACAACC	Jiang *et al.*, 2014
WRKY18-rev	qPCR	TTCTGATGGATGATGGACTG	Jiang *et al.*, 2014
WRKY23-for	qPCR	TGCCATGCCAATGCAAAGGAG	Levée *et al.*, 2009
WRKY23-rev	qPCR	ACCAAAACCCAAAGGCGACAAG	Levée *et al.*, 2009
WRKY70-for	qPCR	AATCCAAGGAGCTACTAC	Jiang *et al.*, 2014
WRKY70-rev	qPCR	GTTACCATTGTTGTTGTGG	Jiang *et al.*, 2014
WRKY89-for	qPCR	TCCAACGATCCACAATAACC	Jiang *et al.*, 2014
WRKY89-rev	qPCR	TAAAACATCACCGCCGTCTC	Jiang *et al.*, 2014
NPR1-for	qPCR	GTTGACCTAAATGAGACACC	Jiang *et al.*, 2014
NPR1-rev	qPCR	TAATCTCAGCCTTGTCCTTG	Jiang *et al.*, 2014
PR1-rev	qPCR	TGGGTTGATGAGAAACCAAAGTATG	Hamel *et al.*, 2011
PR1-for	qPCR	GCTGCACCTTGCTTTAGCAC	Hamel *et al.*, 2011
PR2.3-rev	qPCR	CAAAGGATTGCTTCCAGTCAAGC	Jiang *et al.*, 2014
PR2.3-for	qPCR	TCAAGAAGGGCATCGAAGAGG	Jiang *et al.*, 2014
JAZ10a-for	qPCR	CCCCCTTGACTATTTTCTACAACGG	Hamel *et al.*, 2011
JAZ10a-rev	qPCR	GATCTCCATCAAGACTCTCAAGAAGC	Hamel *et al.*, 2011
MYB115-for	qPCR	GCCATTGGAGGTCTTTGCC	Yoshida *et al.*, 2015
MYB115-rev	qPCR	GGTTACCGAGGAGGGAGTGC	Yoshida *et al.*, 2015
MYB134-for	qPCR	CACCACCACCAATACTGCCAC	Ullah *et al.*, 2017
MYB134-rev	qPCR	CCTGGGCTCTTCAGTTCCG	Ullah *et al.*, 2017
MYB182-for	qPCR	GAATCTTTGGTGACACAGCAAGC	Yoshida *et al.*, 2015
MYB182-rev	qPCR	GAAGCAGAGTTGGCAATGATGA	Yoshida *et al.*, 2015
bHLH131-for	qPCR	GTCGATAATAGAGAGTGACGCA	Yoshida *et al.*, 2015
bHLH131-rev	qPCR	CTCTTCACCTCCACAATGCT	Yoshida *et al.*, 2015
CHS1-for	qPCR	TGTGTGAATACATGGCTCCGTCTCT	Yoshida *et al.*, 2015
CHS1-rev	qPCR	GGATTTTGGCTGACCCCACTCTT	Yoshida *et al.*, 2015
CHS4-for	qPCR	TCACTGTTGAGACTGTGGTG	Wang *et al.*, 2017
CHS4-rev	qPCR	CACTCCTTATTGGTGCTCTC	Wang *et al.*, 2017
CHI1-for	qPCR	TGTGCTAGAGTCAATGATTGG	Wang *et al.*, 2017
CHI1-rev	qPCR	GGAAAAGCTTGAGCCTGAAAT	Wang *et al.*, 2017

F3H1-for	qPCR	TGGTCTGACTTTACAACGTGC	Wang *et al.,* 2017
F3H1-rev	qPCR	GACAACTCACACGGCATTGC	Wang *et al.,* 2017
DFR1-for	qPCR	CTTATAACTGCCCTTTCTCTGA	Yoshida *et al.,* 2015
DFR1-rev	qPCR	AGATCATGAATGGTGGCTT	Yoshida *et al.,* 2015
PnLAR1-for	qPCR	CGAGTACTCATAGCCGGAGC	Ullah *et al.,* 2017
PnLAR1-rev	qPCR	GGCTCCTTTGTCGTGAAGAG	Ullah *et al.,* 2017
PnLAR2-for	qPCR	AACAAGTCGGTCCATTTTCG	Ullah *et al.,* 2017
PnLAR2-rev	qPCR	GCA GCAATAGCAAGGAGGTC	Ullah *et al.,* 2017
PnLAR3-for	qPCR	GAAGCTAGCCTCGAATGTGG	Ullah *et al.,* 2017
PnLAR3-rev	qPCR	TTGGTCTGCTATGCTTGCAC	Ullah *et al.,* 2017
PnANR1-for	qPCR	GCATCCCAGACCAAGAAAAA	Ullah *et al.,* 2017
PnANR1-rev	qPCR	TCCCCCAAATTCTGTAGTGC	Ullah *et al.,* 2017
PnANR2-for	qPCR	CCTGCCTCCAAGACACTAGC	Ullah *et al.,* 2017
PnANR2-rev	qPCR	GCTGCTGGGAATATCTAGCG	Ullah *et al.,* 2017
Ubiquitin-for	qPCR	GTTGATTTTTGCTGGGAAGC	Irmisch *et al.*, 2013
Ubiquitin-rev	qPCR	GATCTTGGCCTTCACGTTGT	Irmisch *et al.*, 2013
MlpActin-for	qPCR	GACTGAGGCACCTCTTAATCCAAAAGTC	Ullah *et al.,* 2017
MlpActin-rev	qPCR	GTGAGTAACACCGTCACCAGAATCC	Ullah *et al.,* 2017
WD40-for	qPCR	CTCGCACGAACCAATACCAG	This study
WD40-rev	qPCR	AACATGAGCTTTGTGGGTGG	This study

References

Hamel LP, Benchabane M, Nicole MC, Major IT, Morency MJ, Pelletier G, Beaudoin N, Sheen J, Seguin A. 2011. Stress-responsive mitogen-activated protein kinases interact with the EAR motif of a poplar zinc finger protein and mediate its degradation through the 26S proteasome. *Plant Physiology* **157**(3): 1379-1393.

Irmisch S, McCormick AC, Boeckler GA, Schmidt A, Reichelt M, Schneider B, Block K, Schnitzler JP, Gershenzon J, Unsicker SB, et al. 2013. Two herbivore-induced cytochrome P450 enzymes CYP79D6 and CYP79D7 catalyze the formation of volatile aldoximes involved in poplar defense. *Plant Cell* **25**(11): 4737-4754.

Jiang Y, Duan Y, Yin J, Ye S, Zhu J, Zhang F, Lu W, Fan D, Luo K. 2014. Genome-wide identification and characterization of the Populus WRKY transcription factor family and analysis of their expression in response to biotic and abiotic stresses. *Journal of Experimental Botany* **65**(22): 6629-6644.

Levee V, Major I, Levasseur C, Tremblay L, MacKay J, Seguin A. 2009. Expression profiling and functional analysis of *Populus* WRKY23 reveals a regulatory role in defense. *New Phytologist* **184**(1): 48-70.

Ullah C, Unsicker SB, Fellenberg C, Constabel CP, Schmidt A, Gershenzon J, Hammerbacher A. 2017. Flavan-3-ols are an effective chemical defense against rust infection. *Plant Physiology* **175**(4): 1560-1578.

Wang L, Ran L, Hou Y, Tian Q, Li C, Liu R, Fan D, Luo K. 2017. The transcription factor MYB115 contributes to the regulation of proanthocyanidin biosynthesis and enhances fungal resistance in poplar. *New Phytologist* **215**(1): 351-367.

Yoshida K, Ma D, Constabel CP. 2015. The MYB182 Protein Down-Regulates Proanthocyanidin and Anthocyanin Biosynthesis in Poplar by Repressing Both Structural and Regulatory Flavonoid Genes. *Plant Physiology* **167**(3): 693-710.

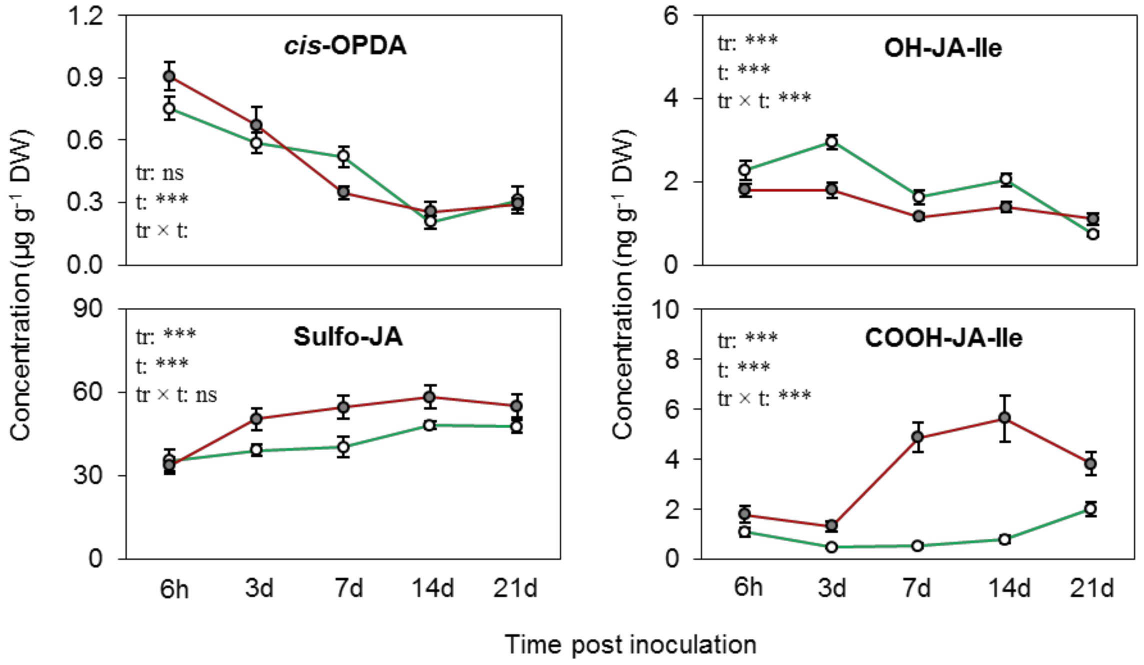

Fig. S1 Effect of rust infection on the concentrations of the jasmonic acid (JA) precursor, *cis*-OPDA, and JA catabolites in black poplar leaves over the course of infection. Rust-infected trees are shown by red lines (closed circles) and non-infected control trees are shown by green lines (open circles). *cis*-OPDA = *cis*-12-oxo-phytodienoic acid, Sulfo-JA = 12-sulfojasmonic acid, OH-JA-Ile = 12-hydroxyjasmonic acid isoleucine, COOH-JA-Ile = 12-carboxyjasmonic acid isoleucine. Data were analyzed by two-way ANOVA. Data are expressed as the mean ± SE (n=5), and each replicate was a pool of six fully expanded leaves (LPI 5-10) from a single tree. tr = treatment; t = time; **, $p \leq 0.01$; and ***, $p \leq 0.001$; ns, non-significant; h = hour, d = day, DW = dry weight.

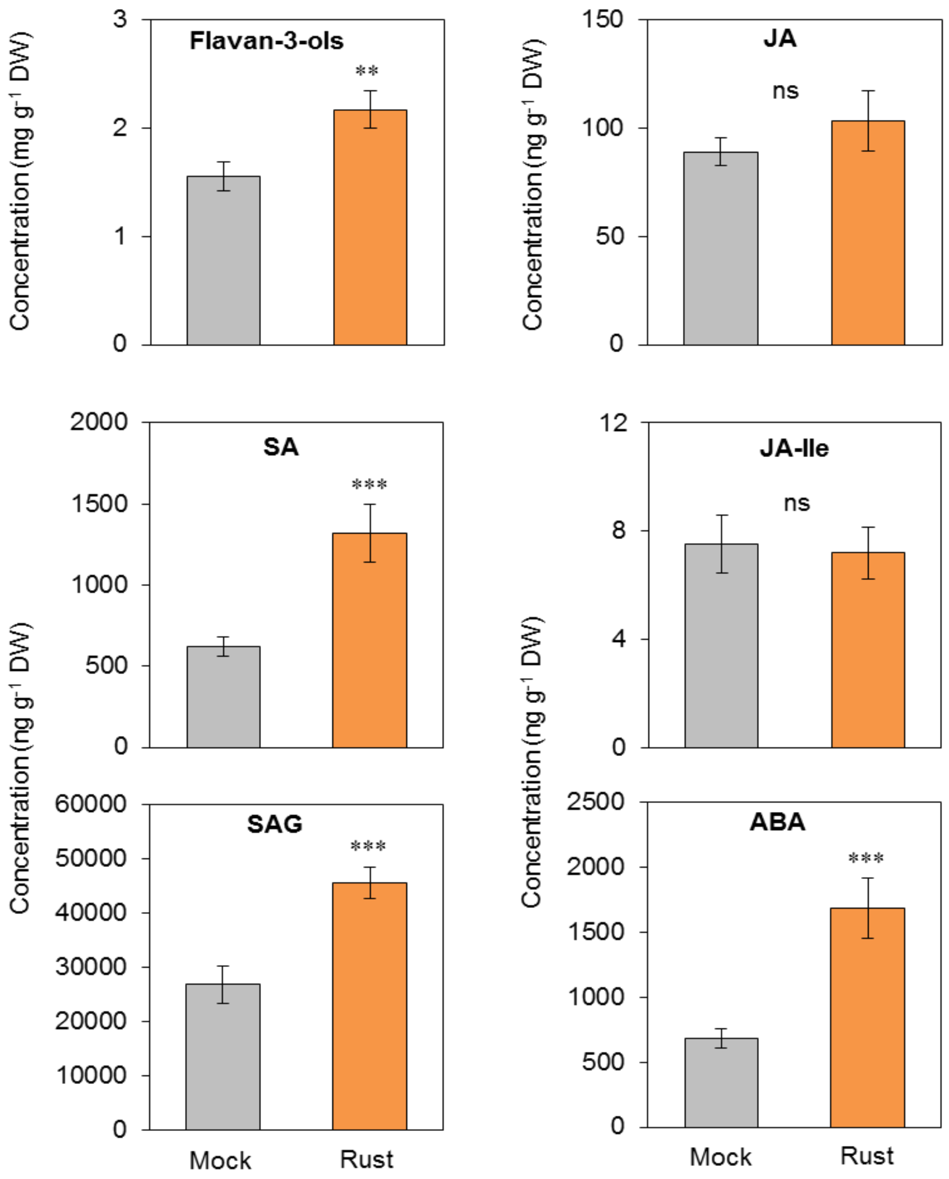

Fig. S2 Effect of rust infection on the accumulation of flavan-3-ols, abscisic acid and salicylic acid in expanding systemic leaves of black poplar trees. Growing shoots (leaf 1-5 counted basipetally) were covered with polyethylene terephthalate (PET) bags before rust inoculation (Rust) or spraying with water (Mock) to protect from fungal infection. Young expanding leaves (LPI 1-5) without rust symptoms were sampled 7 days after inoculation. Flavan-3-ols were measured as the sum of catechin, epicatechin, gallocatechin and proanthocyanidin dimers. SA = salicylic acid, SAG = salicylic acid glucoside, ABA = abscisic acid, JA = jasmonic acid, JA-Ile = jasmonic acid isoleucine, DW = dry weight. Data were analyzed by Student's t-test (**, $p < 0.01$; ***, $p < 0.001$; ns, non-significant). Bars represent mean ± SE (n=5).

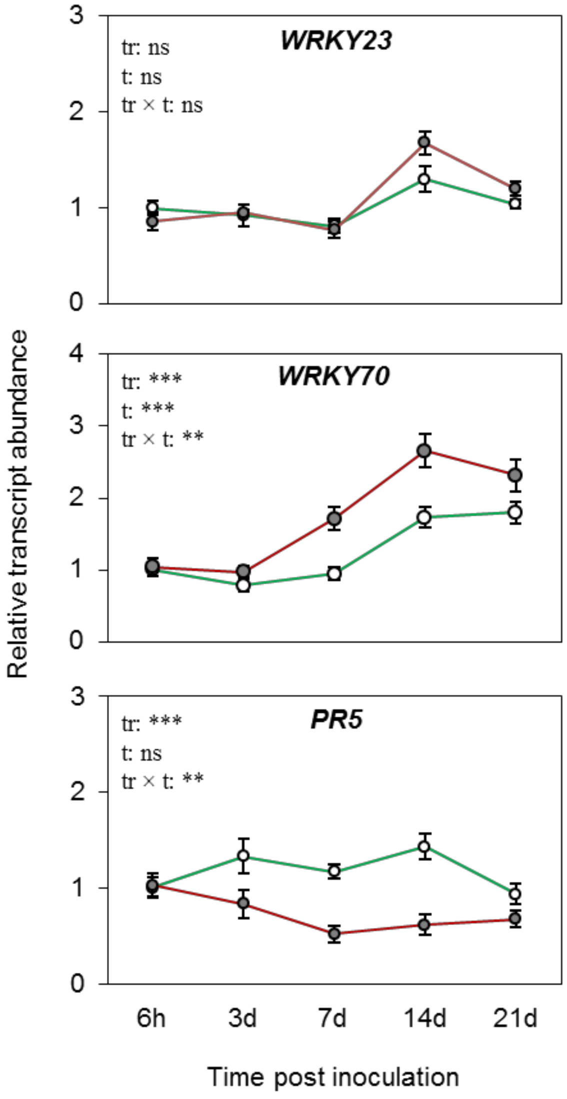

Fig. S3 Effect of rust infection on the relative expression levels of *WRKY23*, *WRKY70* and *PR5*. Rust-infected trees are shown by red lines (closed circles) and non-infected control trees are shown by green lines (open circles). Transcript levels of each gene were normalized to *ubiquitin* transcripts. Three technical replicates were used per sample during qRT-PCR. Data were analyzed by two-way ANOVA (Factors: "tr" = treatment, "t" = time post inoculation). Data are expressed as the mean ± SE (n=5), and each replicate was a pool of six fully expanded leaves (LPI 5-10) from a single tree. tr = treatment; t = time; **, $p \leq 0.01$; and ***, $p \leq 0.001$; ns, non-significant. h = hour; d = day.

Table S2 Levels of phenolic metabolites in black poplar leaves one day after exogenous hormone treatment.

Phenolic metabolites	Treatment			
	Mock	BTH	MeJA	ABA
Catechin (µg/g)	293 ± 21	441 ± 46	350 ± 55	337 ± 58
Epicatechin (µg/g)	19 ± 5.2	28 ± 3.1	22 ± 5.3	19 ± 4.6
Gallocatechin (µg/g)	46 ± 8.3	50 ± 4.1	66 ± 14.7	39 ± 4.0
PAB1 (µg/g)	91 ± 11	124 ± 17	89 ± 15	95 ± 19
Salicinoids (mg/g)	34 ± 1.2	31 ± 1.4	33 ± 1.6	33 ± 1.7
Phenolic acids (µg/g)	8.3 ± 0.40 a	6.0 ± 0.39 b	6.8 ± 0.31 ab	8.1 ± 0.42 a
Rutin (mg/g)	5.1 ± 0.97	5.9 ± 0.87	5.4 ± 0.81	5.5 ± 0.79

Leaf laminae (LPI 5-10) from benzothiadiazole (BTH), methyl jasmonate (MeJA), abscisic acid (ABA) and mock-treated young black poplar trees were analyzed one day after treatment, just before rust inoculation (0 dpi). PAB1 = procyanidin B1. Salicinoids were the sum of salicin, salicortin and homaloside D. Phenolic acids were the sum of cinnamic acid, coumaric acid, caffeic acid and ferulic acid. Data were analyzed by one-way ANOVA followed by Tukey's Post-hoc test. Different letters indicate groups were statistically different ($p < 0.05$). Data are presented as the mean ± SE (n=4).

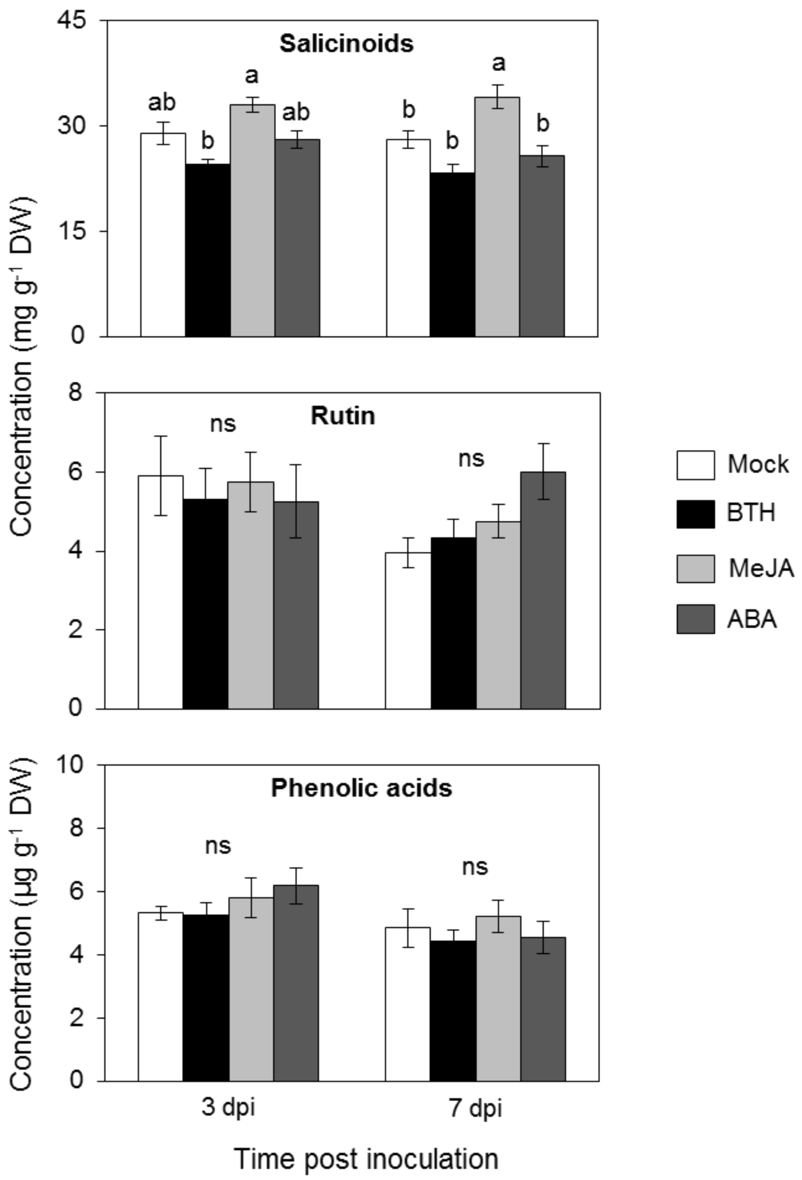

Fig. S4 Phytohormone application before rust infection did not alter the levels of other phenolic metabolites in poplar besides flavan-3-ols. Salicinoids were the sum of salicin, salicortin and homaloside D. Phenolic acids were the sum of cinnamic acid, coumaric acid, caffeic acid and ferulic acid. Samples were collected from separate trees at each time point for all treatments. Time-course data were analyzed by one-way ANOVA followed by Tukey's Post-hoc test. Different letters indicate groups were statistically different ($p < 0.05$). Data are presented as the mean ± SE (n=5), and each replicate was a pool of six fully expanded leaves (LPI 5-10) from a single tree. dpi = day post inoculation of rust fungus, ns = non-significant, DW = dry weight.

Table S3 Levels of endogenous hormones in black poplar leaves one day after exogenous hormone treatment.

Metabolites	Treatment			
	Mock	BTH	MeJA	ABA
SA (ng/g)	325 ± 76 ab	213 ± 38 b	649 ± 129 a	517 ± 71 a
JA (ng/g)	70 ± 14 b	50 ± 12 b	1970 ± 141 a	82 ± 14 b
JA-Ile (ng/g)	5.1 ± 0.9 b	5.7 ± 0.7 b	34.7 ± 4.5 a	5.8 ± 0.8 b
cis-OPDA (ng/g)	517 ± 55 b	425 ± 43 b	1295 ± 69 a	274 ± 25 c
OH-JA (ng/g)	498 ± 70 b	362 ± 46 b	2962 ± 594 a	275 ± 44 b
OH-JA-Ile (ng/g)	3.89 ± 0.38 b	3.15 ± 0.43 b	44.79 ± 5.84 a	4.07 ± 0.63 b
COOH-JA-Ile (ng/g)	1.34 ± 0.40 b	1.74 ± 0.39 b	10.13 ± 0.78 a	1.77 ± 0.28 b
ABA (ng/g)	79 ± 15 c	76 ± 20 c	349 ± 78 b	37236 ± 3287 a

Leaf laminae (LPI 5-10) from benzothiadiazole (BTH), methyl jasmonate (MeJA), abscisic acid (ABA) and mock-treated young black poplar trees were analyzed one day after treatment, just before rust inoculation (0 dpi). SA = salicylic acid, JA = jasmonic acid, JA-Ile = jasmonic acid-isoleucine conjugate, *cis*-OPDA = *cis*-12-oxo-phytodienoic acid, OH-JA = 12-hydroxyjasmonic acid, COOH-JA-Ile = 12-carboxyjasmonic acid-isoleucine, OH-JA-Ile = 12-hydroxyjasmonic acid-isoleucine. Data were analyzed by one-way ANOVA followed by Tukey's Post-hoc test. Different letters indicate significant differences between treatments ($p < 0.05$). Data are presented as the mean ± SE (n=4).

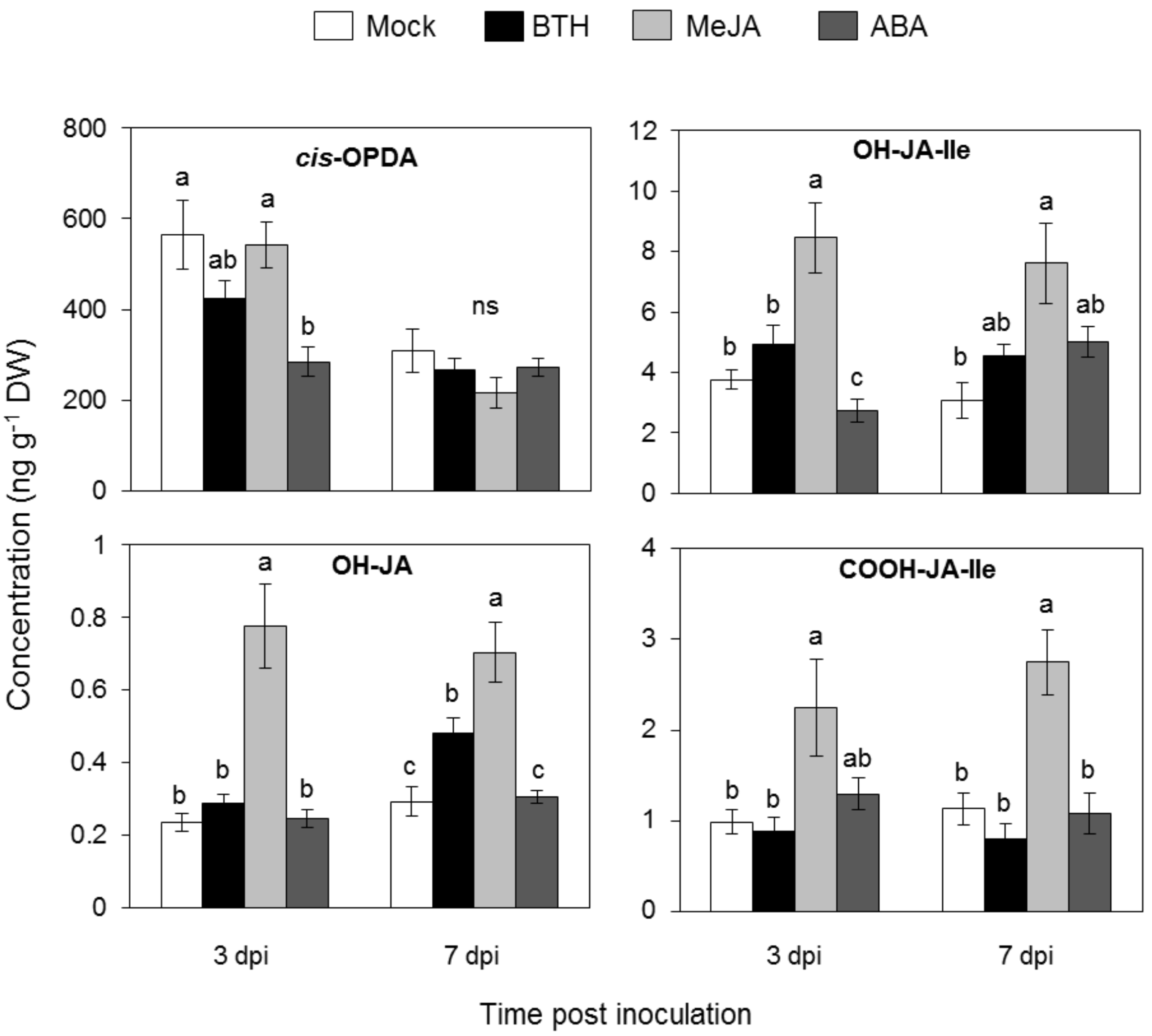

Fig. S5 Jasmonate concentrations in *Populus nigra* leaves after exogenous phytohormone application followed by rust infection. *cis*-OPDA = *cis*-12-oxo-phytodienoic acid, OH-JA = 12-hydroxyjasmonic acid, OH-JA-Ile = 12-hydroxyjasmonic acid-isoleucine, COOH-JA-Ile = 12-carboxyjasmonic acid-isoleucine. Samples were collected from separate trees at each time point for all treatments. Time-course data were analyzed by one-way ANOVA followed by Tukey's Post-hoc test. Different letters indicate groups were statistically different at 95% confidence. Data are presented as the mean ± SE (n=5), and each replicate was a pool of six fully expanded leaves (LPI 5-10) from a single tree. dpi = day post inoculation of rust fungus; ns = non-significant, DW = dry weight.

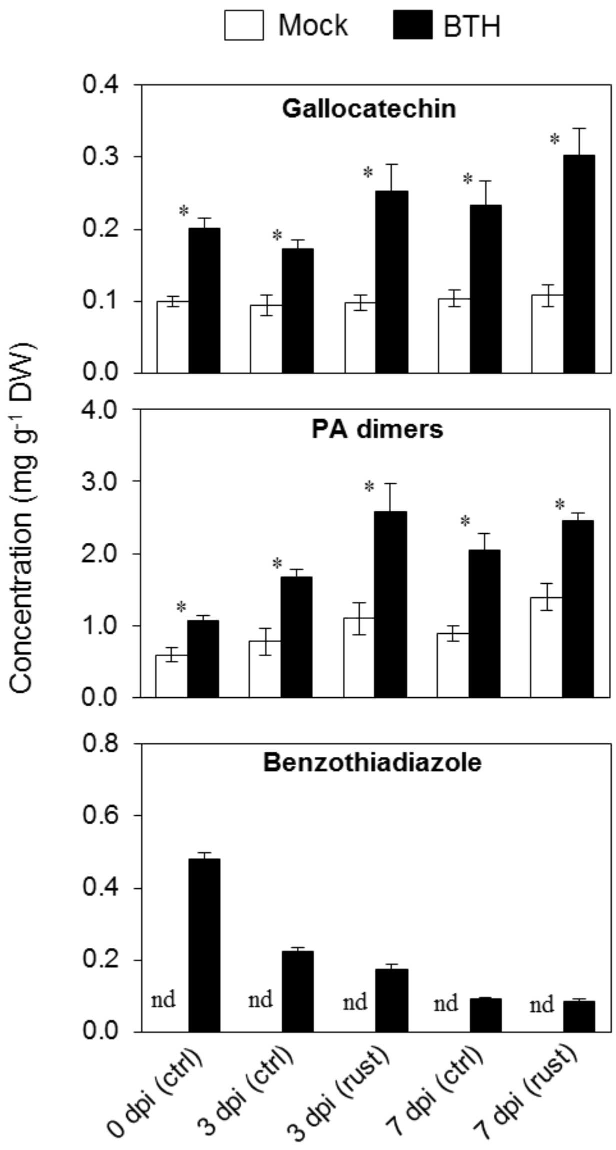

Fig. S6 Accumulation of flavan-3-ols in black poplar leaves treated with the salicylic acid analogue benzothiadiazole. Data (mock *vs* BTH) were analyzed by Student's t-test (*, $p < 0.05$). Data are presented as the mean ± SE (n=4), and each replicate was a pool of six fully expanded leaves (LPI 5-10) from a single tree. PA = proanthocyanidins, dpi = day post inoculation, ctrl= water-treated control, rust = rust-inoculated, nd = not detected, DW = dry weight.

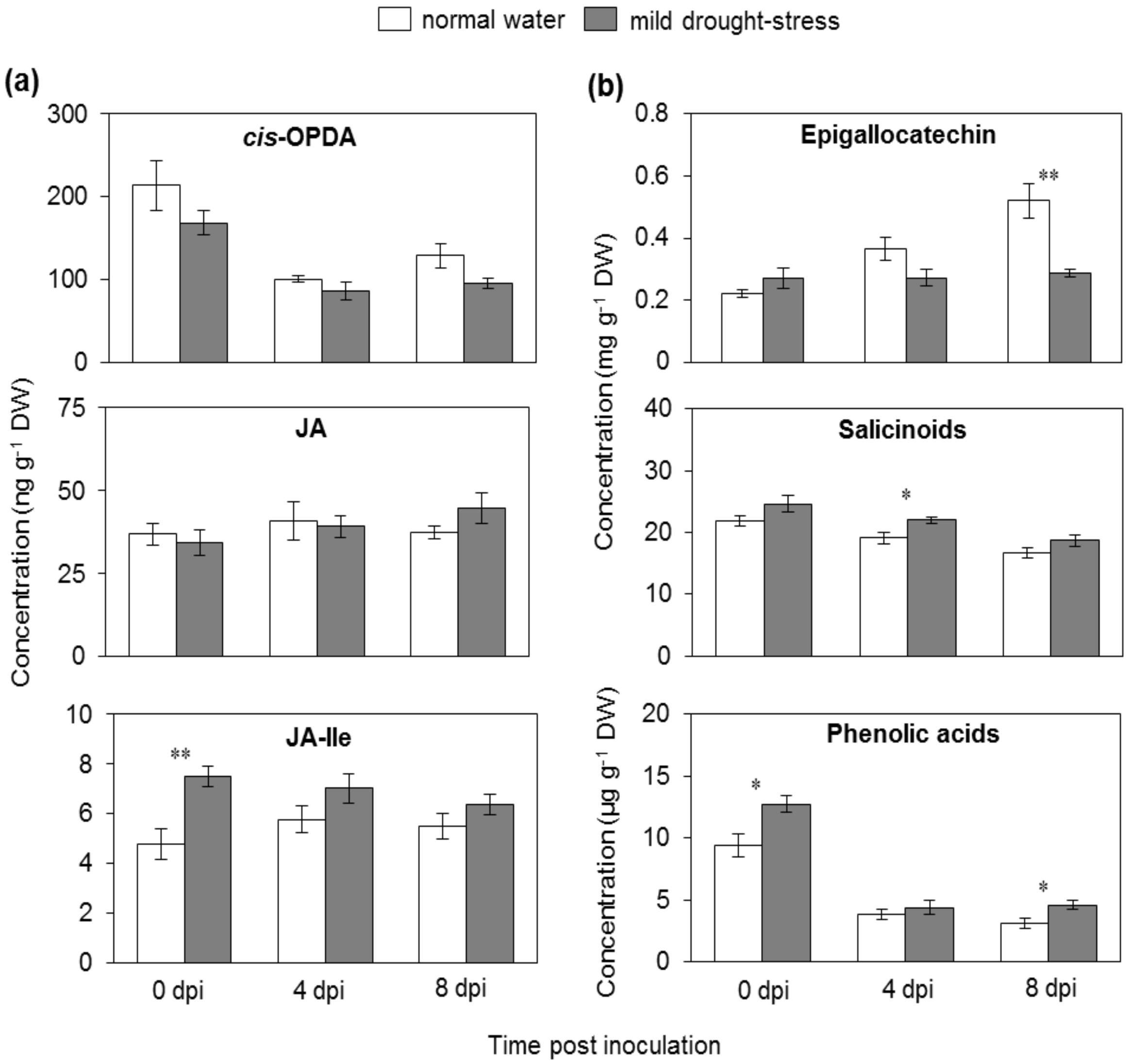

Fig. S7 Concentrations of jasmonates, epigallocatechin, salicinoids and phenolic acids in poplar (*Populus* × *canadensis* Robusta) leaves under mild drought stress and rust infection. Young poplar trees were watered normally or exposed to mild drought stress. After 7 days, a subset of plants was sampled (0 dpi) and the remaining trees were inoculated with *M. larici-populina*. The drought treatment was continued until the end of the experiment. *cis*-OPDA = *cis*-12-oxo-phytodienoic acid, JA = jasmonic acid, JA-Ile = jasmonic acid isoleucine. Salicinoids were the sum of salicin, salicortin and homaloside D. Phenolic acids were the sum of cinnamic acid, coumaric acid, caffeic acid and ferulic acid. Data were analyzed by Student's t-test (*, $p < 0.05$ and **, $p \leq 0.01$). Data are presented as the mean ± SE (n=5), and each replicate was a pool of five fully expanded leaves (LPI 5-10) from a single tree. dpi = days post inoculation, ns = non-significant, DW = dry weight.
